# One-Channel Surface Electromyography Decomposition for Muscle Force Estimation

**DOI:** 10.3389/fnbot.2018.00020

**Published:** 2018-05-04

**Authors:** Wentao Sun, Jinying Zhu, Yinlai Jiang, Hiroshi Yokoi, Qiang Huang

**Affiliations:** ^1^Intelligent Robotics Laboratory, School of Mechatronical Engineering, Beijing Institute of Technology, Beijing, China; ^2^Beijing Innovation Center for Intelligent Robots and Systems, Beijing, China; ^3^Intelligent Control Laboratory, College of Engineering, Peking University, Beijing, China; ^4^School of Informatics and Engineering, University of Electro-Communications, Tokyo, Japan; ^5^Key Laboratory of Biomimetic Robots and Systems, Ministry of Education, Beijing, China

**Keywords:** sEMG decomposition, reconstruction independent component analysis, motor unit action potentials, grip force estimation, prosthetic hand control

## Abstract

Estimating muscle force by surface electromyography (sEMG) is a non-invasive and flexible way to diagnose biomechanical diseases and control assistive devices such as prosthetic hands. To estimate muscle force using sEMG, a supervised method is commonly adopted. This requires simultaneous recording of sEMG signals and muscle force measured by additional devices to tune the variables involved. However, recording the muscle force of the lost limb of an amputee is challenging, and the supervised method has limitations in this regard. Although the unsupervised method does not require muscle force recording, it suffers from low accuracy due to a lack of reference data. To achieve accurate and easy estimation of muscle force by the unsupervised method, we propose a decomposition of one-channel sEMG signals into constituent motor unit action potentials (MUAPs) in two steps: (1) learning an orthogonal basis of sEMG signals through reconstruction independent component analysis; (2) extracting spike-like MUAPs from the basis vectors. Nine healthy subjects were recruited to evaluate the accuracy of the proposed approach in estimating muscle force of the biceps brachii. The results demonstrated that the proposed approach based on decomposed MUAPs explains more than 80% of the muscle force variability recorded at an arbitrary force level, while the conventional amplitude-based approach explains only 62.3% of this variability. With the proposed approach, we were also able to achieve grip force control of a prosthetic hand, which is one of the most important clinical applications of the unsupervised method. Experiments on two trans-radial amputees indicated that the proposed approach improves the performance of the prosthetic hand in grasping everyday objects.

## Introduction

As the basic driver of human locomotion, muscle force is an important evaluation index in research on and treatment of biomechanical diseases, disabilities, disorders, and injuries. Invasive measurement of muscle force can cause pain or injury to the subject (Connan et al., 2016), and the used of non-invasive techniques is therefore desirable. Surface electromyography (sEMG), using biopotentials recorded on the skin, provides a window on muscle states and has become one of the most popular non-invasive techniques for estimating muscle force (Höppner et al., 2017).

Methods for estimating muscle force by sEMG fall into two types: supervised and unsupervised. In the supervised method, sEMG signals and the corresponding force measured by additional devices are recorded to tune variables of the mapping from sEMG to the force, for example coefficients of linear regression (Hoozemans and Van Dieen, 2005), weights of an artificial neural network (Liu et al., 2017), and parameters of model-based algorithms (Hayashibe and Guiraud, 2013). Although it can achieve high accuracy when estimating muscle force, this method requires a large dataset to tune multiple variables and additional devices to record the force. In addition, it is challenging to apply the supervised method to conditions where recording of the force is impossible, a particular case being the recording of muscle force from lost limbs of amputees. In the unsupervised method, muscle force recording is not needed. Nevertheless, this method suffers from low accuracy of force estimation due to a lack of reference data.

The conventional unsupervised method estimates muscle force as being proportional to the sEMG amplitude (Hoozemans and Van Dieen, 2005). It has been discovered, however, that the relation between sEMG amplitude and muscle force is linear only at low force levels (Solomonow et al., 1986). For high force levels, the relation becomes non-linear owing to extensive superimposition of sEMG signals (McGill, 2004). Superimposition of sEMG signals means that positive and negative action potentials from different motor units (MUs) cancel each other, with a consequent reduction in the energy of the summed sEMG signals. In addition, the conventional method treats sEMG signals as numerical data, without any consideration of muscle physiology. All the factors that affect the EMG-force relationship are concentrated in a one-dimensional signal. This imperfect one-dimensional signal, affected by factors irrelevant to force production, is neither precise nor representative for force estimation (Staudenmann et al., 2010).

A muscle is composed of hundreds to thousands of MUs—the basic units that produce muscle force. This force can be increased by increasing the number of activated MUs and their firing rates (Sandbrink and Ellad, 2016). The activated MUs generate motor unit action potentials (MUAPs) and the summation of the MUAPs is the sEMG signal. By decomposing sEMG signals into their constituent MUAPs, superimpositions of sEMG signals can be avoided to a large extent. Therefore, theoretically, muscle force can be estimated more accurately from the constituent MUAPs than from one-dimensional sEMG.

To decompose an sEMG signal, independent component analysis (ICA) is commonly adopted. This approach, which assumes linear independence of the firing pattern of MUAPs (Blok et al., 2002), has been shown to excel in separating sEMG signals with several overlapped MUAPs (Comon and Jutten, 2010). ICA is commonly applied using high-density electrodes (Holobar et al., 2012; Farina et al., 2014, 2015), because large-scale data are required to ensure its performance (Deng et al., 2012). However, high-density electrodes require expensive recording instruments and involve complex operations. This limits the applicability of ICA to real-time muscle force estimation.

In this paper, we propose an unsupervised approach for accurate muscle force estimation with one-channel sEMG. This approach decomposes a one-channel sEMG signal into its constituent MUAPs and estimates muscle force based on these MUAPs. First, the approach learns an orthogonal basis of sEMG signals through reconstruction independent component analysis (RICA), a transformed ICA with a soft form of constraint (Le et al., 2011). The use of RICA instead of ICA is able to achieve decomposition of one-channel sEMG signals with a considerably reduced number of electrodes. Second, MUAPs are extracted from basis vectors and form clusters according to their shape. The centers of the clusters are memorized as the most representative MUAPs. The learned basis is made sparse by replacing spikes in the basis vector with the most representative MUAPs and by padding the rest of the basis vector with zeros. This process of obtaining a sparse matrix is called the training process. The next process is testing. During this, the sparse matrix obtained from the training process is used to decompose new sEMG signals in real time. Because of the sparsity of the matrix, its inverse can be computed rapidly to obtain the firing rate and the number of activated MUs from sEMG. Muscle force is estimated in proportion to the firing rate and the number of activated MUs.

To compare the accuracy of the proposed approach with that of the conventional approach to muscle force estimation, experiments were carried out on nine healthy subjects. Both sEMG and muscle force were recorded to evaluate the accuracy. In addition, we applied the proposed approach to control the grip force of a prosthetic hand—one of the most important clinical applications of the unsupervised method (Riillo et al., 2014). Grasping experiments on two trans-radial amputees were carried out to evaluate the improvements in performance of the prosthetic hand in grasping everyday objects.

## sEMG model

In this section, a widely accepted sEMG model composed of MUAPs and noise is introduced. MUAPs are modeled as sparse and periodic spikes to meet the sparsity and linear independence assumptions of RICA. The sEMG signal is composed of a mixture of firing patterns of MUAPs recorded by surface electrodes during muscle contraction. Other factors such as sweat, subject movement, and interference from the electrical mains supply produce noise in sEMG. The mixing in the sEMG signal can be represented as

(1) $$\begin{array}{l} x=As+\;\varepsilon \end{array}$$

The sEMG signal *x* is a linear combination of the firing patterns of MUAPs *s* multiplying by a mixing matrix *A* and the Gaussian noise ε. Each column of *A* represents the passive volume conduction effect of the biological tissue between the electrodes and the MUs (Clark and Plonsey, 1966; Farina et al., 2004). These volume conduction effects largely determine the features of the detected sEMG signal in terms of its shape and frequency content (Staudenmann et al., 2010). The mixing matrix *A* is a time-varying variable, because the volume conductor is affected by various factors such as subject movement and sensor displacement. However, *A* can be treated as stationary over a period of time in which changes in *A* happen rather slowly. In addition, biologists have shown that the nervous system increases the strength of muscle contraction by increasing the number and the firing rate of activated MUs, without changing the features of the signal (Sandbrink and Ellad, 2016). MUAPs are transformed spikes from the nervous system, and they share common properties in terms of their shape and probability of occurrence (Byrne and Dafny, 2015). Biological findings have revealed two properties of MUAPs: one is the stationarity of MUAP features at all force levels, and the other is the sparsity and independent occurrence of MUAPs. In a period of time *T*, the mixing in the sEMG signal can be expressed as

(2) $$\begin{array}{l} x\left(t\right)=\;\sum_{j=1}^{L}\sum_{d=0}^{T-1}a_{j}s_{j}\left(t-d\right)+\;\varepsilon \left(t\right),\;t=0,\;1,..,T, \end{array}$$

where *L* is the number of MUs, *d* is the time shift, *a*_*j*_ is the filtering effect of tissues between MU *j* and the electrode, and *s*_*j*_(*t*) is the firing pattern of MU *j*. Equation (2) indicates that the sEMG signal can be treated as a linear combination of time-shifted MUAPs. RICA and *K*-means were used to separate MUAPs from the sEMG signals. The details of the decomposition will be presented in the next section.

## sEMG signal decomposition and force estimation

**Figure 1:**
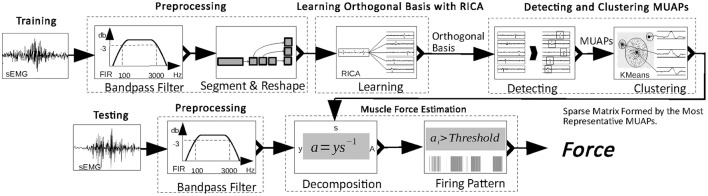
Workflow of the proposed muscle force estimation approach.

In this section, an approach to the decomposition of the sEMG signal into its constituent MUAPs and an estimation of the muscle force based on MUAPs is proposed. Figure 1 shows the workflow of the proposed approach. Two main processes are involved: the training process and the testing process. In the training process, the raw sEMG signal is filtered and reshaped. Then, from the reshaped sEMG signal, an orthogonal basis is learned. Finally, MUAPs are extracted from basis vectors and form clusters with the most representative MUAP chosen as the center of each cluster. In the testing process, the sEMG signal is multiplied by a sparse matrix formed from the most representative MUAPs to obtain the firing patterns of the MUAPs. Muscle force is estimated in proportion to the number of activated MUs and the firing rate. The details of the process are described in the following subsections.

### Preprocessing

The raw sEMG signal is sampled at 16 kHz and preprocessed in two steps. First, it is filtered by a band-pass filter (100–3,000 Hz, 3 dB) to remove noise. Then the filtered signal is standardized with zero mean and unit variance. The standardized sEMG is reshaped to a *m*×*n* matrix to avoid singularity in decomposing the sEMG signal. In the remainder of this paper, the sEMG signal refers to data passed through this two-step preprocessing, and is denoted by *x*.

### Learning the basis of sEMG with RICA

ICA is an algorithm for learning an orthogonal basis from data. However, ICA is time-consuming, because the orthogonal constraint is non-differentiable. To slightly loosen the constraint and speed up learning, the orthogonal constraint of ICA is replaced with a soft reconstruction loss and a sparsity penalty. This soft form of ICA is referred to as RICA, which reduces calculation time and is thus a practical tool for real-time data decomposition.

The model introduced in Section sEMG Model assumes that sEMG is composed of sparsely occurring and linearly independent MUAPs that are spikes in the orthogonal basis vectors of the sEMG signal. The key issue in learning the basis is to find a transform matrix *W* that minimizes the cost function *J*(*W*). The cost function of ICA constrains basis vectors to be mutually orthogonal. RICA penalizes the sparsity and minimizes the reconstruction loss, and thus guarantees that the basis vector is as orthogonal as possible (Le et al., 2011). The method assumes *W* to be of full column rank to avoid singularities in matrix manipulation. Therefore, it is necessary to learn a basis with *k* basis vectors of *x*, *m* ≥ *k*. The cost function *J*(*W*) used by RICA to decompose the sEMG signal is

(3) $$\begin{array}{l} J\left(W\right)=\;\lambda ||Wx|{|}_{1}+\frac{1}{2}||W^{T}Wx-x|{|}_{2} \end{array}$$

Here λ is the penalty factor (λ = 0.01); || ||_1_is the absolute difference, which constrains the sparsity of the basis; and || ||_2_ is the square difference, which measures the differences between the original and the reconstructed sEMG signal. The optimum *W* can be quickly calculated with a gradient descent optimizer. *x* is decomposed into *k* basis vectors *b*_*i*_ (*i* = 1, 2, …, *k*) by the transform matrix *W*:

(4) $$\begin{array}{l} {\left[b_{1},\ldots ,b_{i},\ldots ,b_{k}\right]}^{T}=Wx \end{array}$$

Note that *b*_*i*_ is composed of the spike-like MUAPs and noise. The approach to detecting MUAPs from the basis vectors and clustering the MUAPs will be introduced in the next subsection.

### Detecting and clustering MUAPs

An amplitude threshold is set to detect MUAPs from the basis learned with RICA. The amplitude threshold is set at 4σ to split signal from background noise:

(5) $$\begin{array}{l} \sigma =\text{median}\left(\frac{\left|x\right|}{0.6745}\right) \end{array}$$

where |*x*| returns the absolute value of all elements of *x*. σ gives a fast and accurate estimation of background noise (Donoho and Johnstone, 1994). Choosing the median instead of the standard deviation of data avoids high threshold values when MUs are at high firing rate and large amplitude (Quiroga et al., 2004).

Spikes in the basis vectors *b*_*i, i* = 1, 2, …, *k*_ with absolute peak values larger than the threshold are assumed to be MUAPs. For each spiky MUAP, 100 samples (6.25 ms) are extracted from the basis vector with the peak value at sample 50. Furthermore, MUAPs with peak value less than zero are flipped to avoid misalignment of the MUAP. If the MUAP is flipped, the corresponding column in *W* will be multiplied by −1 to avoid changing the sign of *x*. MUAPs *y*_*i*_ are extracted from the basis vector *b*_*i*_ according to the rule

(6) $$\begin{array}{l} y_{i}=\{\begin{array}{c} sgn\left(peak\left(b_{i}\right)\right)*spike\left(peak\left(b_{i}\right),\;100\right),\;\left|peak\left(b_{i}\right)\right|>4\sigma ,\\ 0,\;otherwise, \end{array} \end{array}$$

where *sgn* is the sign function, *peak*(*b*_*i*_) returns the peak of *b*_*i*_, and *spike*(*peak*(*b*_*i*_), 100) returns the 100 datapoints from *b*_*i*_ aligned to the peak. MUAPs are clustered by the *K*-means algorithm in terms of their shape. The shape of an MUAP is measured according to its Fourier coefficients.The *K*-means algorithm minimizes the within-cluster sum of squares (Arthur and Vassilvitskii, 2007) and has been used in a wide range of applications (Kuo et al., 2005). *K*-means has one drawback in that the number of clusters has to be provided in advance. For clustering MUAPs, the number of clusters, set from 5 to 20, reflects the maximum number of MUs potentially detected by a one-channel electrode. The number of MUs can be chosen to be <5 or more than 20, but doing so will lead to inconsistencies with biological findings with regard to the firing rate and interpulse interval of the MUs. The optimum number of clusters is searched by Grid Search, which uses a scoring system based on the silhouette metric. This metric is employed to model the distribution of the cluster and to find the optimal balance between tightness and separation (Rousseeuw, 1987). The score is defined as

(7) $$\begin{array}{l} score=\;\frac{\sum_{l=1}^{L}\left(sc_{l}\;>\;\bar{sc}\right)}{L} \end{array}$$

where $\bar{sc}$ is the average silhouette coefficient for all samples and *sc*_*l*_ is the average silhouette coefficient for the sample in cluster *l. L* is the number of clusters. The number of clusters with the highest score is chosen as the optimum one. After the number of clusters has been determined, the *K*-means algorithm is applied. The *K*-means algorithm classifies MUAPs into *l* clusters. The center of cluster *l* is utilized as the most representative MUAP of the cluster, which is denoted by *r*_*l*_. All MUAPs in cluster *l* are replaced by *r*_*l*_:

(8) $$\begin{array}{l} s_{i}=\{\begin{array}{c} b_{i}\left[replace\left(y_{i},\;r_{l}\right)\right],\;kmeans\left(y_{i}\right)=l,\\ 0,\;otherwise, \end{array} \end{array}$$

where *s* is a sparse form of the basis *b*, *b*_*i*_[*replace*(*y*_*i*_, *r*_*l*_)] replaces 100 data points in *b*_*i*_ from which *y*_*i*_ has been extracted with *r*_*l*_, and *kmeans*(*y*_*i*_) returns the cluster label of *y*_*i*_. The inverse of *s* can be approximated by LSQR (Paige and Saunders, 1982), which allows fast decomposition of the sEMG signal. In the following subsection, an *s*^−1^-based approach is proposed to estimate the muscle force.

### Muscle force estimation and evaluation

This subsection presents an approach to estimating muscle force from the firing rate of MUAPs and the number of activated MUs. Research has proved that muscle force has a positive correlation with the number of active MUs and their firing rates—the frequency at which the MU is activated (Sandbrink and Ellad, 2016). To calculate the firing rate, we count the number of MUAPs in 0.05 s. For any sEMG signal in 0.05 s, $\bar{x}\;\left(1\times n\right)$, the data are treated as linear combination of the sparse matrix *s*:

(9) $$\begin{array}{l} \left[{\bar{a}}_{1},{\bar{a}}_{2},\ldots ,{\bar{a}}_{k}\right]=\bar{x}s^{-1}, \end{array}$$

where $\left[{\bar{a}}_{1},{\bar{a}}_{2},\ldots ,{\bar{a}}_{k}\right]$ is called the coordinate of $\bar{x}$ with respect to *s*. If the absolute value of ${\bar{a}}_{i}$ is greater than the constant *c* (here *c* = 0.34), then we assume that the MU of cluster *l* corresponding to the *i*th row of *s* is activated. In fact, ${\bar{a}}_{i}$ denotes the strength of the constituent *s*_*i*_ in $\bar{x}$. If the strength is greater than *c*, then the MU corresponding to *s*_*i*_ is assumed to be activated. The number of activated MUs is calculated by counting the number of activated clusters. The muscle force $\overset{ˇ}{\text{F}}$ is calculated by

(10) $$\begin{array}{l} \overset{ˇ}{F}=\phi \frac{\sum_{i=1}^{k}\left(|{\bar{a}}_{i}|>c\right)}{k}unique\left(kmeans\left(y_{i},if\;|{\bar{a}}_{i}|>c\right)\right) \end{array}$$

where ϕ is a scaling coefficient and *unique*() returns the number of unique elements; for example, *unique*([1, 2, 2, 3]) = 3 owing to the duplicated 2 in the vector. For an sEMG signal in a period of 0.5 s, the muscle force is calculated by the above equation.

The estimated force $\overset{ˇ}{\text{F}}$ is evaluated with *R*-squared analysis:

(11) $$\begin{array}{l} R^{2}=\left(1-\frac{var\left(\overset{ˇ}{F}-F\right)}{var\left(F\right)}\right)\times 100\% \end{array}$$

where *F* is the measured force, and the function *var*(*z*) returns the variance of the variable *z*. When *R*^2^ = 1, the estimated force explains all the variability of the measured force around its mean, and when *R*^2^ = 0, the estimated force explains none of the variability of the measured force around its mean.

## Experiments and results

### Experimental setup

To evaluate the accuracy of our force estimation approach, we carried out experiments on nine healthy subjects—seven males (subjects 1, .., 7, aged 30.0 ± 5.6 years) and two females (subjects 8 and 9, aged 25.0 ± 0.2 years)—after they had signed informed consent documents. The subjects reported no history of upper-extremity or other musculoskeletal disorders.

**Figure 2:**
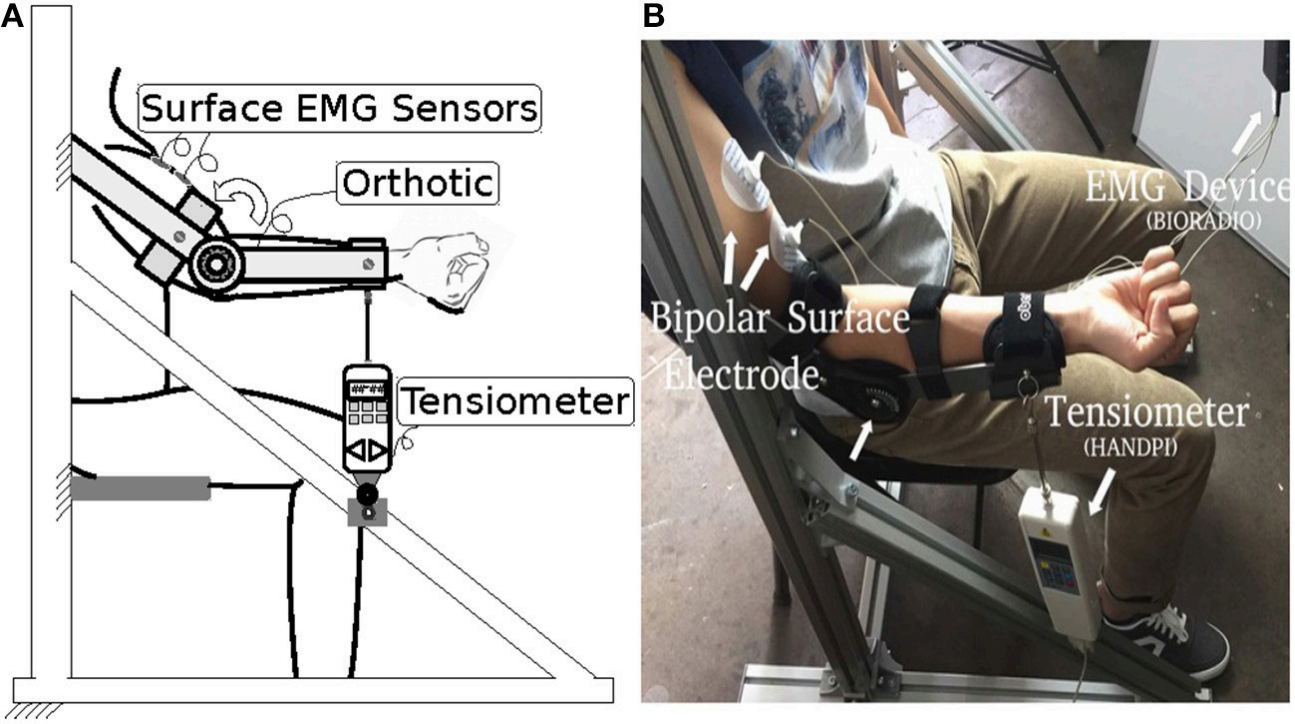
Experimental setup **(A)** diagrammatic side view; **(B)** photograph.

The experiment setup is shown in Figure 2. A bipolar electrode (Ag–AgCl) was attached to the biceps brachii of the subject. sEMG was performed using a portable sEMG device (BioRadio®) at 16 kHz, and muscle force was recorded by a tensiometer (Handpi®) at 10 Hz. Each subject was instructed to sit still in a chair with his or her right arm fastened to an upper-limb orthosis. The orthosis kept the forearm in the horizontal plane and only allowed the elbow joint to flex. The tensiometer was connected to the end of the orthosis. In the experiment, the subjects flexed their right arms and maintained a comfortable level force for one second and then relaxed for one more second. The subjects were asked to repeat this procedure eight times. The data from the first three times were used as the trainset and the data from the remaining five times as the testset.

### Experimental results and discussion

Sections Extracting MUAPs from the Learned Basis to Reconstructing sEMG Signals from MUAPs present the experimental results of the training process, and Section Accuracy of Force Estimation discusses the accuracy of the approach to force estimation during the testing process.

#### Extracting MUAPs from the learned basis

**Figure 3:**
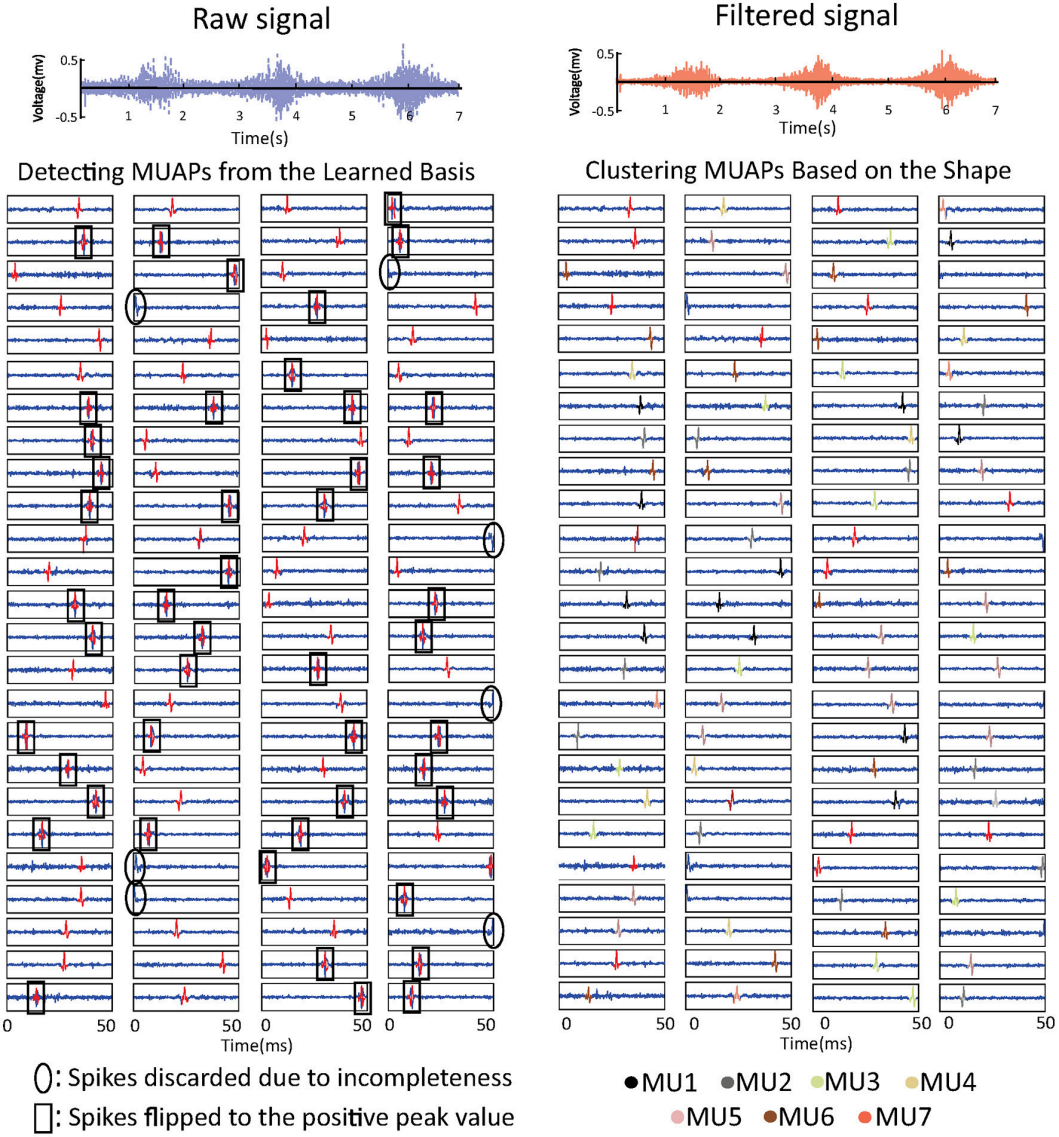
sEMG signal decomposition: the raw sEMG signal (top left), the bandpass-filtered signal (top right), the detected MUAPs from the learned basis vectors (bottom left), and the clustered MUAPs (bottom right).

The learned basis is shown in Figure 3. The raw sEMG signal was filtered, segmented, and reshaped into *x*_*m*×*n*_ (*m* = 120, *n* = 800), then *b*_*k*_ (*k* = 100) basis vectors were learned from *x*_*m*×*n*_ based on Equation (3). In the figure, basis vectors are colored blue and the spike-like MUAPs are colored red. One can observe that 93 MUAPs were extracted from basis vectors based on the amplitude threshold 4σ. Spikes in basis vectors of width <100 samples, marked with black ovals in the figure, were discarded owing to incompleteness. Spikes with negative peak values, which are marked with black squares, were flipped. The 93 MUAPs were clustered by the *K*-means algorithm into seven groups (MU1, …, MU7) based on their shape. MUAPs in the same cluster were highlighted in the same color, as shown on the right side of Figure 3.

In order to learn the basis reliably and quickly, several assumptions on the basis were made, including the sparsity of MUAPs and the linear independence of the firing patterns of the MUs. Although these assumptions have not been rigorously proved, acceptable mathematical results were obtained. Any sEMG signal in 0.05 s is expressed as a linear combination of orthogonal basis vectors that contain firing patterns of MUs in 0.05 s. In theory, for muscle force estimation, the use of large basis vectors should improve the accuracy of force estimation. On the other hand, however, large basis vectors undermine the real-time performance of the proposed approach.

#### Shape of MUAPs

**Figure 4:**
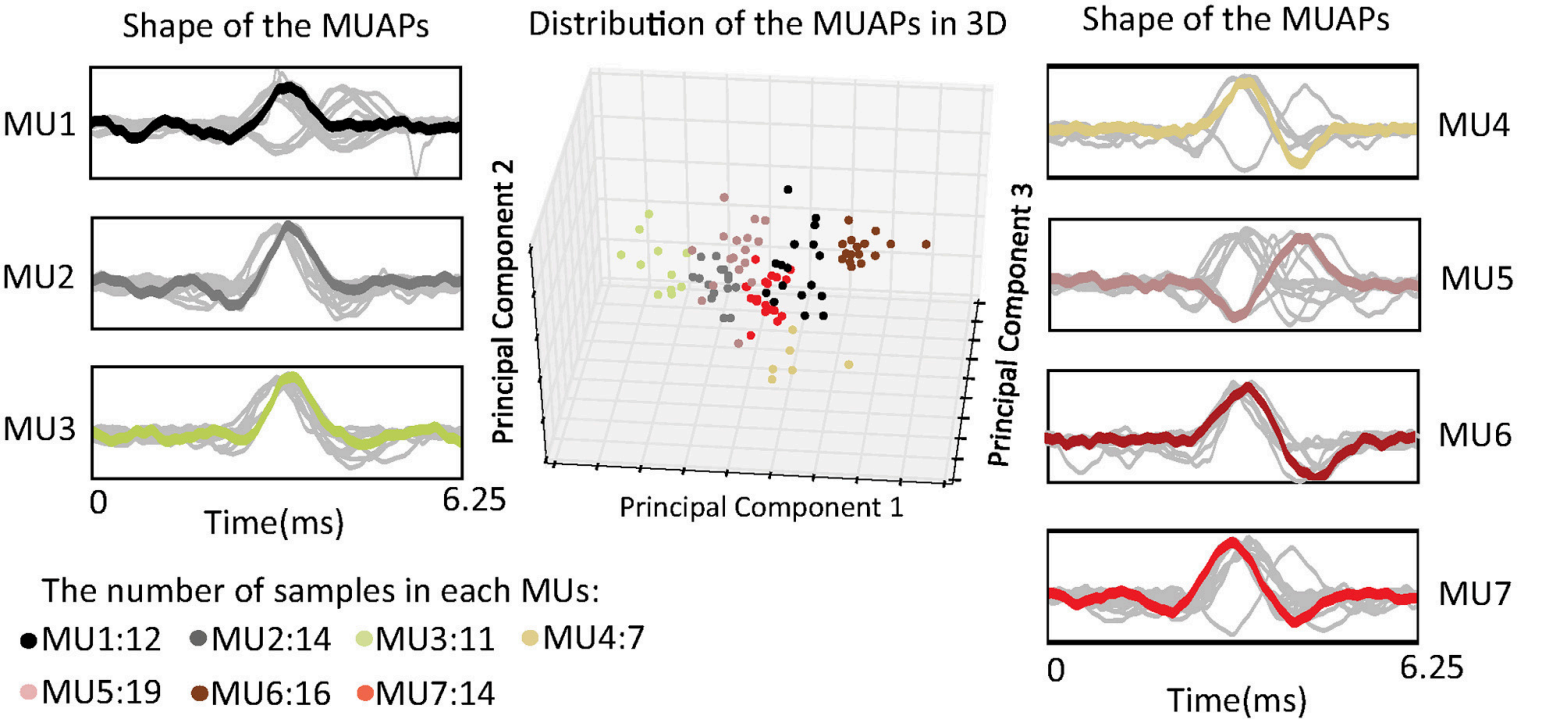
Distribution of the shape of the MUAPs in three dimensions using principal component analysis.

MUAPs of the same cluster have similar shapes, and they are assumed to be generated by the same MU. Figure 4 shows distributions of MUAP shape through principal component analysis (PCA) and in three dimensions. The distance between any two points represents a measurement of their differences in shape as given by Fourier coefficients. From the figure, one can observe that the distances between different samples in the same cluster are almost equal. This can probably be attributed to the fact that the silhouette metric models the data distribution as a Gaussian and prefers clusters with equal variance (the same tightness). The most representative MUAP of each cluster, i.e., the center of the cluster, is colored, while the remaining MUAPs are gray.

Clustering of MUAPs determines the number of MUs detected in muscle. In Equation (10), the number of MUs provides the non-linearity in force estimation. Removing this non-linearity will reduce the accuracy of the proposed approach.

#### Firing rates of MUs

**Figure 5:**
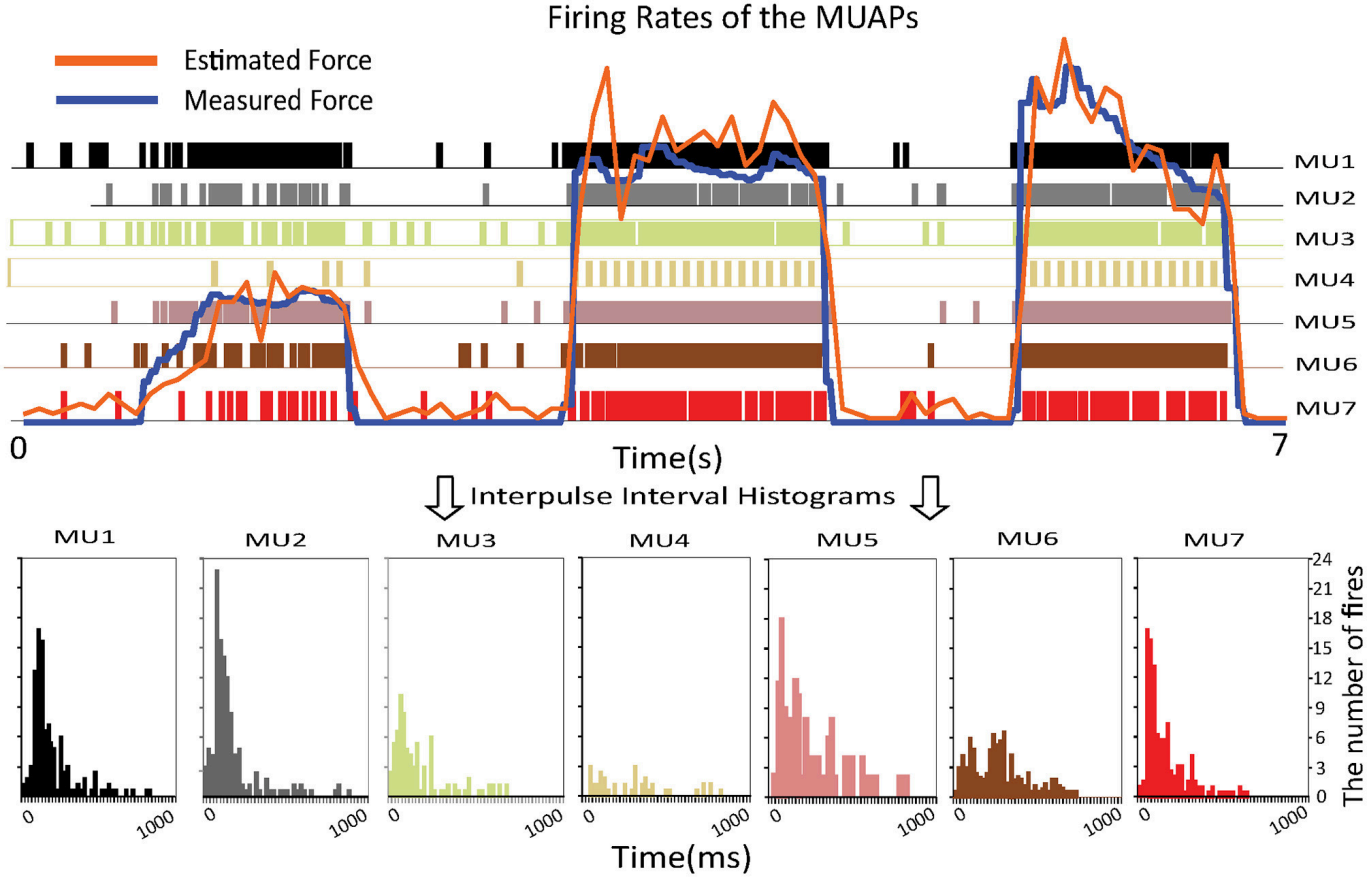
Relationship between the firing rates of the MUAPs and the muscle force **(Top)** and interpulse interval histograms of the MUs **(Bottom)**.

The upper part of Figure 5 shows the estimated and measured forces. The colored vertical bars of unit height indicate the firing pattern of a specific MU. The densities of these bars represent the firing rates. One can observe that the firing rate of the MUs increases as the muscle force rises. To prove that the proposed approach is consistent with biological findings, interpulse interval histograms of the MUs are shown at the bottom of the figure. Most of the MUs fired with interpulse intervals centered around 50–100 ms, which means that the firing rates of the MUs are around 10–20 Hz. These histograms agree with results on the statistical properties of the interpulse interval (Stock and Thompson, 2016), which shows the feasibility of the proposed approach.

#### Reconstructing sEMG signals from MUAPs

By decomposing an sEMG signal into MUAPs, the part of the signal irrelevant to force production is removed. Reconstructing sEMG signals from MUAPs is a direct way of observing the signals after decomposition. The reconstructed signal $\overset{ˇ}{x}$ can be obtained as

(12) $$\begin{array}{l} \overset{ˇ}{x}=W^{-1}s. \end{array}$$

**Figure 6:**
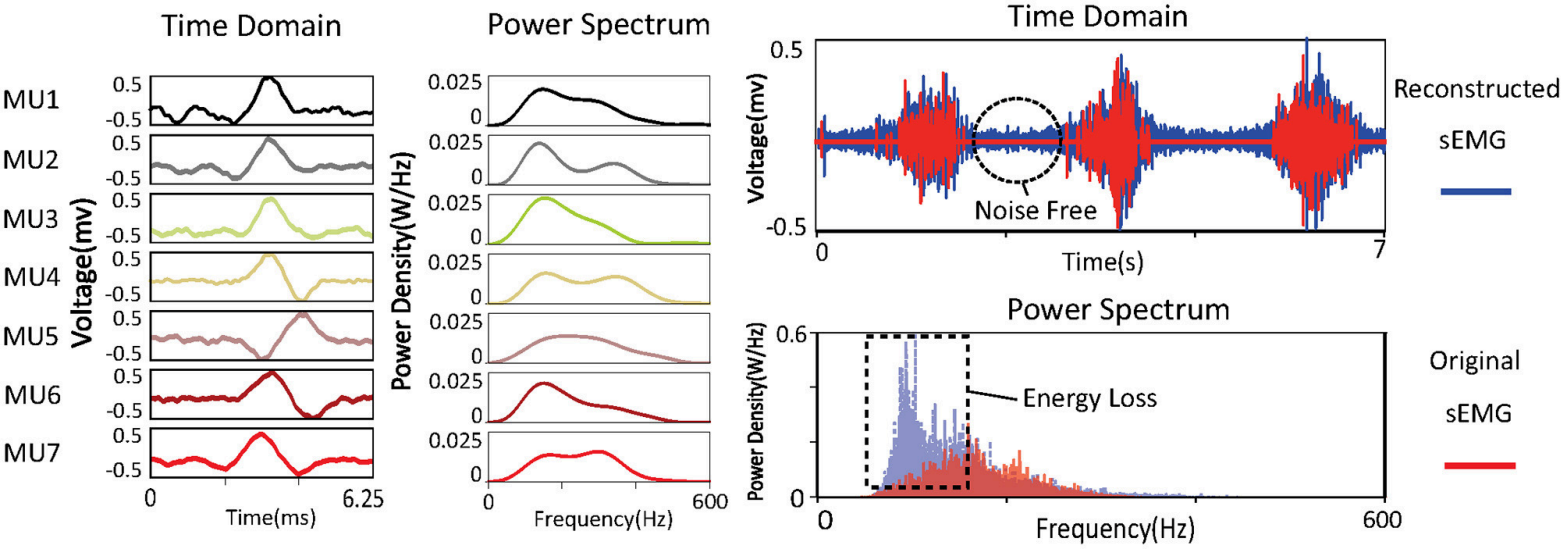
Time domain and power spectra of MUAPs and sEMG signals.

Power spectra of the decomposed MUAPs are shown on the left of Figure 6. The energy loss between the original and the reconstructed sEMG signals can probably be attributed to the spike-like shapes of MUAPs. The power spectra of MUAPs are bell-shaped, with centers around 200 Hz. The reconstructed sEMG signal can be regarded as the raw sEMG after passage through a non-linear filter. The shapes and power spectra of the original and reconstructed sEMG signals are shown on the right of Figure 6. The reconstructed signal is similar to the original, except that the noise in the latter has been removed. Although the two signals look alike in the time domain, their power spectra differ significantly. The power spectrum of the original sEMG signal is strongly concentrated around 100 Hz, but that of the reconstructed signal is centered around 200 Hz, which is consistent with the results of a study of the power spectra of MUAPs (Tanzi and Taglietti, 1981). The reconstructed sEMG signal retains 56% of the energy of the original signal.

#### Accuracy of force estimation

**Figure 7:**
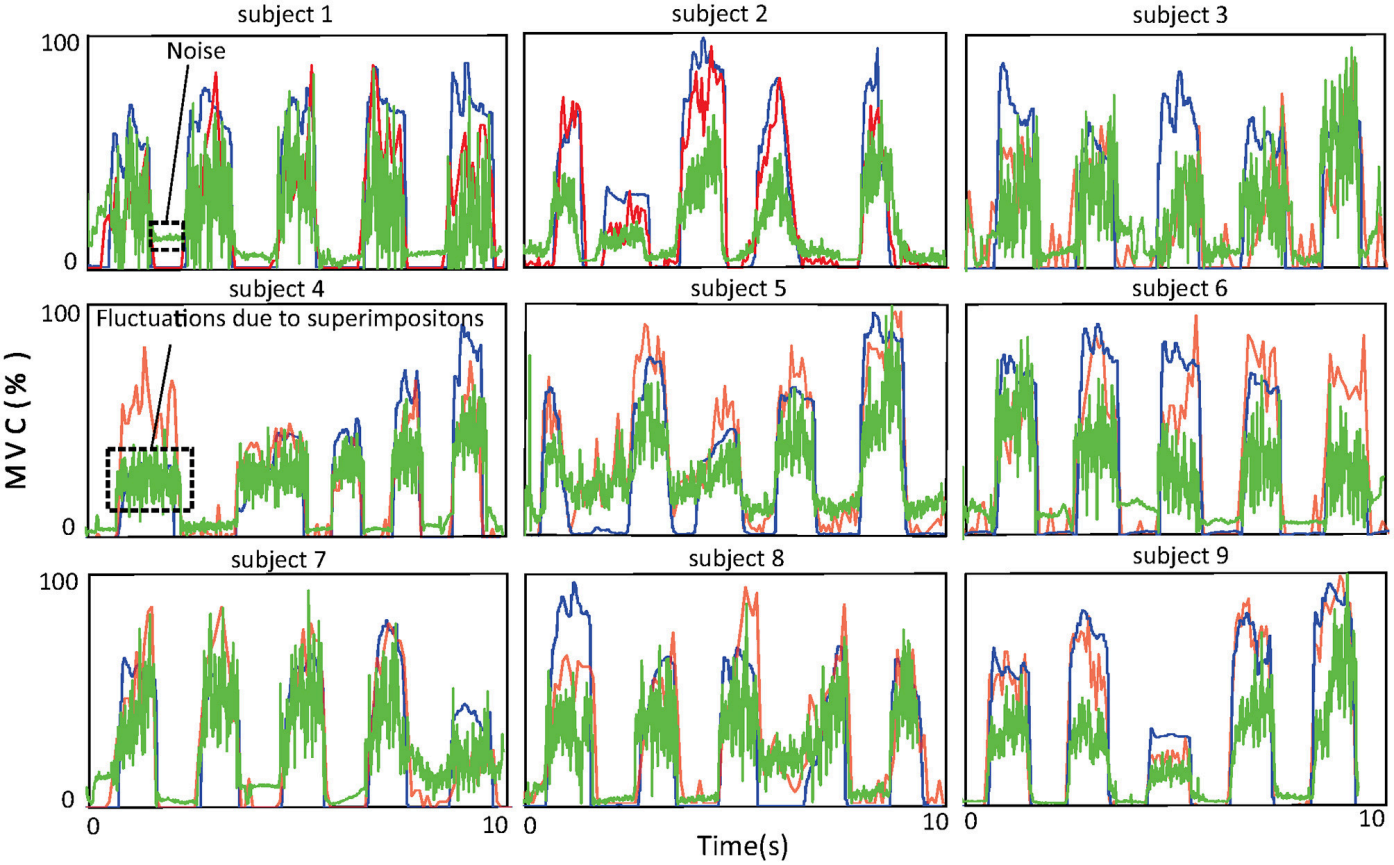
Force curves for the nine subjects. The blue curves show the force recorded by the tensiometer, the red curves the force estimated by the proposed approach, and the green curves the force estimated from the amplitude of the sEMG signal.

The accuracy of force estimation for the nine healthy subjects is shown in Figure 7. One thing that deserves attention here is that muscle force is normalized with respect to the maximal voluntary contraction (MVC) of the subject. The blue, red, and green lines denote the force recorded by the tensiometer, the force estimated by the proposed approach, and the force estimated from the amplitude of the sEMG signal, respectively. The *R*^2^ values of the force estimated by the proposed approach with respect to the recorded force (subjects 1, …, 9) are 72.6, 87.3, 88.1, 72.2, 76.3, 80.2, 81.3, 84.6, and 88.9%. The *R*^2^ values of the force estimated from the amplitude with respect to the recorded force (subjects 1, …, 9) are 67.3, 43.1, 61.3, 63.5, 57.5, 68.2, 68.9, 62.7, and 68.3%. The average *R*^2^ value of the proposed approach is 81.3% ± 6.1% and is superior to that of the approach based on the amplitude (62.3% ± 7.7%). The improvement in performance achieved with the proposed approach can probably be attributed to its robustness to superimposition of sEMG signals and to noise. From the figure, one can observe that at low force levels, where noise surpasses the sEMG signal, the approach based on the amplitude wrongly predicts a force even when none is produced. At high force levels, superimpositions of sEMG lead to severe fluctuations in the force estimated by the approach based on the amplitude, the reason for this being that the energy of the sEMG signal ceases to increase with rising muscle force.

The accuracy of muscle force estimation confirms the validity of the decomposition. The force can be estimated according to the biological rule stating that muscle force is proportional to the number of activated MUs and their firing rates. If the decomposed MUAPs had been inconsistent with the biological findings on MUAPs, then the muscle force estimated according to the biological rule would have diverged from the force recorded by the tensiometer. Moreover, we believe that the accuracy of the proposed approach can be improved if the parameters involved are fine-tuned for each subject.

## Application to a prosthetic hand

The experimental results on healthy subjects show that the proposed approach performs well in estimating muscle force. To further evaluate the performance of this approach in a clinical application, we applied it to the grip force control of our lab-made prosthetic hand.

### Prosthetic hand control

**Figure 8:**
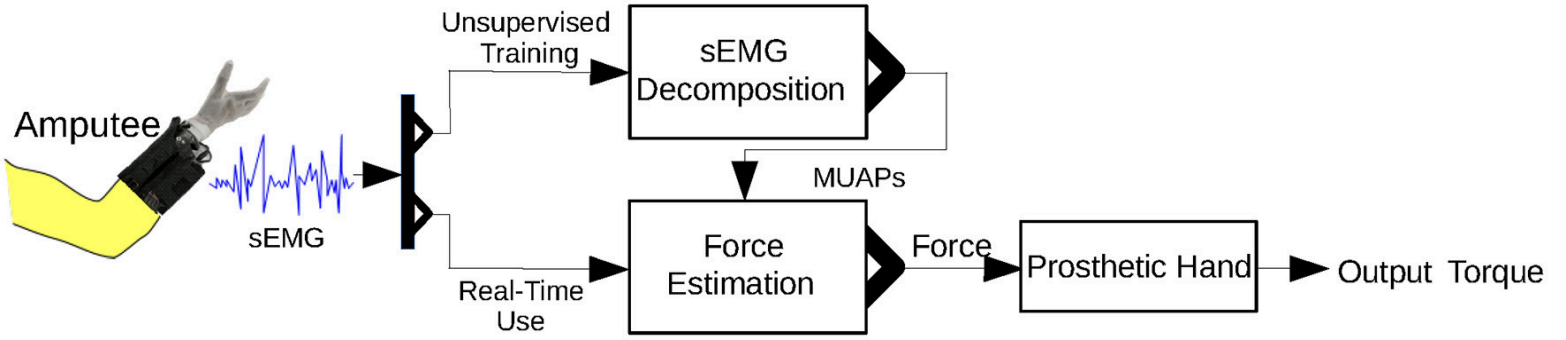
Control strategy for the prosthetic hand using the proposed approach. Unsupervised training is required before real-time use of the prosthetic hand.

A diagram of the control strategy is shown in Figure 8. Because the proposed approach is unable to run entirely on a microcontroller, we downloaded the inverse of the sparse matrix *s*^−1^ to a microcontroller that runs only Equations (9) and (10). The microcontroller thereby achieves real-time muscle force estimation.

An unsupervised training process is required before the amputee can use the prosthetic hand. In this training process, the amputee is instructed to perform the MVC. There are two main reasons for this: first, the MVC activates most of the MUs in a muscle and hence allows detection of a maximal number of MUAPs; second, the MVC can be used to normalize the muscle force. *s*^−1^ is calculated from the sEMG signal of the MVC.

### Grasping experiments

**Figure 9:**
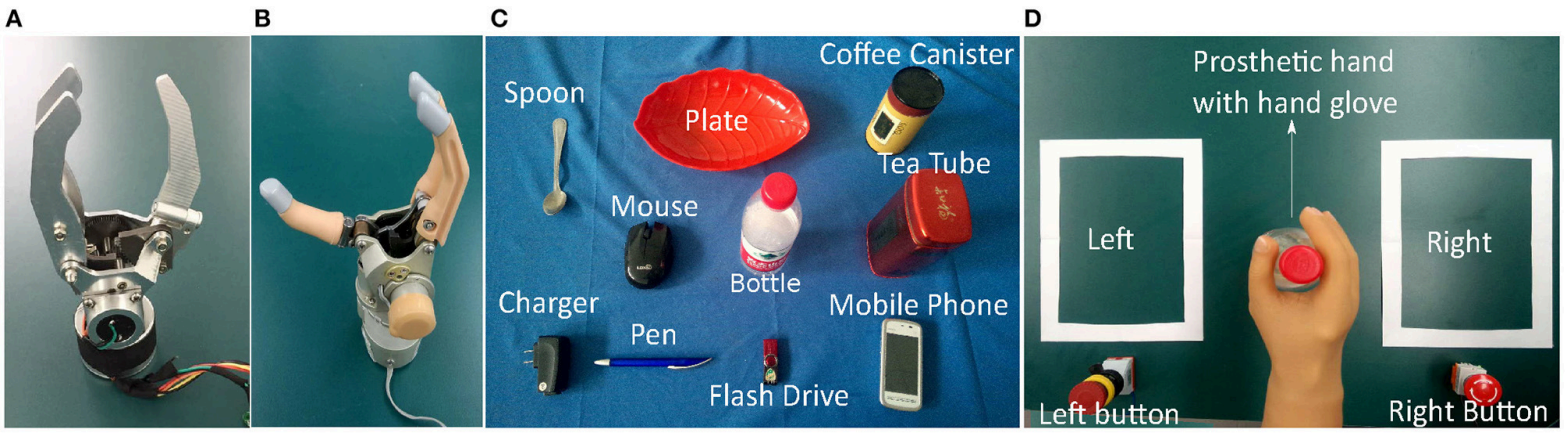
Setup of the grasping experiment: **(A)** lab-made prosthetic hand without hand glove; **(B)** Kesheng hand without hand glove; **(C)** everyday objects for grasping. **(D)** snapshot of grasping experiment.

Grasping experiments were conducted to compare our lab-made prosthetic hand (Figure 9A) controlled by the proposed approach with a commercial prosthetic hand from Kesheng, a Chinese prosthetic hand company (Figure 9B). Both hands have one degree of freedom. Their weights and shapes are almost the same, but their transmission mechanisms are quite different. The Kesheng hand is controlled by motor torque delivered in proportion to the amplitude of the sEMG signal. In the grasping experiment, both prosthetic hands wore a five-finger rubber glove; the ring finger and little finger of both prosthetic hands are passive.

Two trans-radial amputees (subject A aged 34 and subject B aged 45), after signing informed consent documents, participated in the grasping experiments. Subject A has 3 years' experience and Subject B 6 months' experience using the Kesheng hand. sEMG sensors were integrated in the socket and placed on different residual muscles of the subject. The amputees were asked to grasp everyday objects both with our lab-made prosthetic hand and with the Kesheng hand. As shown in Figure 9C, objects for grasping included a spoon, a mobile phone, a tea tube, a coffee canister, a flash drive, a bottle, a plate, a pen, a computer mouse, and a charger. The procedures for each grasping experiment were as follows (Figure 9D): (1) the amputee picked up an object from the left white square and placed it in the right white square; (2) after successfully doing this, the amputee pushed the right red button and earned a score of 1; (3) the amputee then picked up the object from the right square and put it back in the left square; (4) after successfully doing this, the amputee pushed the left red button and earned another score of 1. Each amputee was asked to repeat procedures (1), …, (4) to get as high a total score as possible in 1 min.

## Results and discussion

**Figure 10:**
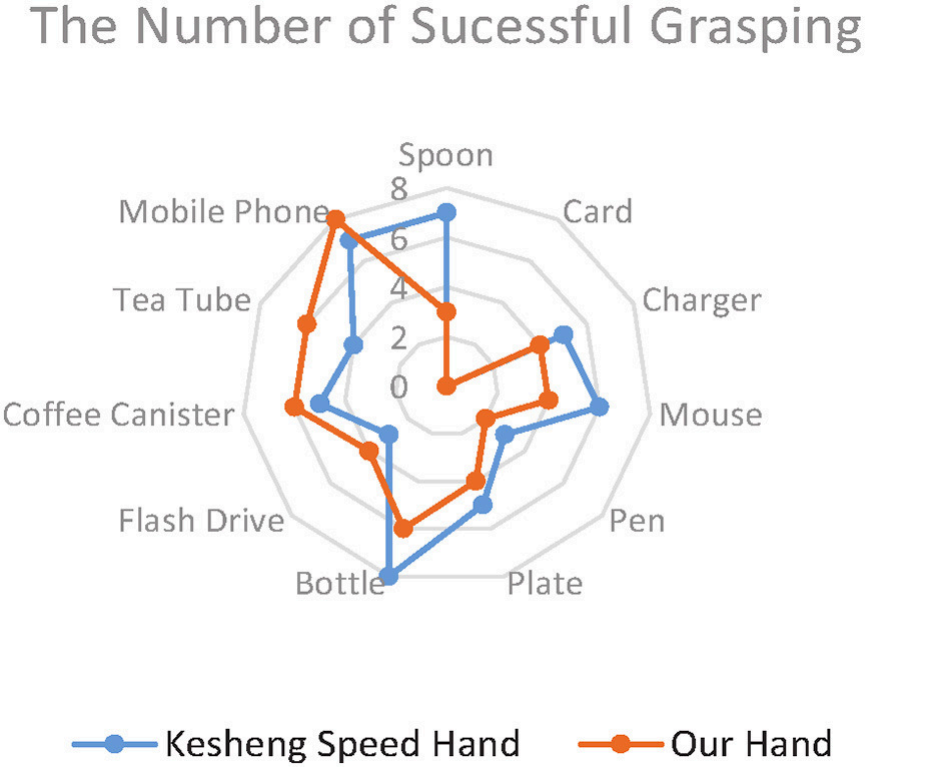
Average scores of the prosthetic hands in the grasping experiments.

Figure 10 shows the average scores for the two prosthetic hands in grasping everyday objects. The total score of our lab-made hand (53) is 11.3% higher than that of the Kesheng hand (47). The performances of the two hands did not differ much in grasping objects of regular shape, such as the mobile phone (8, 7), the coffee canister (6, 5), and the bottle (7, 8).However, when it came to irregularly shaped objects, such as the spoon and plate, our lab-made prosthetic hand achieved much higher scores (7/8) than the Kesheng hand (3/1). This huge gap in scores implies that our proposed approach outperforms the approach based on the amplitude of sEMG signals. Our lab-made prosthetic hand can control the grip force accurately for fast and stable grasping of irregularly shaped objects. In contrast, the grip force of the Kesheng hand is prone to fluctuations caused by noise in the sEMG signal, and the contact force between the hand and the object dramatically changes. These problems result in failure of grasping.

## Conclusion

We have proposed an accurate force estimation approach using one-channel sEMG and have applied it to force control of a lab-made prosthetic hand. Experimental results on nine healthy subjects indicate that this approach is more accurate than the conventional amplitude-based approach. In addition, the results of grasping experiments on two trans-radial amputees demonstrate that with the proposed approach our lab-made hand achieves more stable and faster grasping than a commercial hand. In the future, we aim to investigate the effects of segmentation interval widths of sEMG signals and of RICA redundancies on the accuracy of force estimation, as well as applying this approach to different prostheses.

## Ethics statement

This study was carried out in accordance with the recommendations of the Local Ethics Committee of Peking University (Beijing, China) with written informed consent from all subjects. All subjects gave written informed consent in accordance with the Declaration of Helsinki. The protocol was approved by the Local Ethics Committee of Peking University (Beijing, China).

## Author contributions

WS and JZ: proposed the decomposition approach and drafted the manuscript; YJ, HY, and QH: conducted the experiments and analyzed the data.

### Conflict of interest statement

The authors declare that the research was conducted in the absence of any commercial or financial relationships that could be construed as a potential conflict of interest. The reviewers YL and HW declared a shared affiliation, with no collaboration, with the authors to the handling Editor.

## Supplementary material

The Supplementary Material for this article contains the python code for the proposed algorithm and can be found online at: https://www.frontiersin.org/articles/10.3389/fnbot.2018.00020/full#supplementary-material
