# Supplementary material for: One-Channel Surface Electromyography Decomposition for Muscle Force Estimation
Source: Front Neurorobot. 2018 May 4;12:20. doi: 10.3389/fnbot.2018.00020 (PMC5945831; doi:10.3389/fnbot.2018.00020)
Supplement: Supplementary file 1 [file Image_1.PDF]

sEMG_Decomposition_for_neurorobotics

December 10, 2017

In [1]:
```
#dependency
from __future__ import print_function
from __future__ import division
import numpy as np
np.set_printoptions(threshold=np.nan)
import matplotlib.pyplot as plt
import matplotlib
import scipy.io as sio
import csv
import time
import os
from scipy import signal
import matplotlib.patches as mpatches
```

In [2]:
```
%matplotlib inline
```

In [3]:
```
figDir = 'fig'
rawData = []
with open('rawData.csv', 'rb') as f:
    reader = csv.reader(f)
    for row in reader:
        rawData.append(row[1])

rawData = np.array(rawData[1:], dtype=float)

#
#rawData = rawData[50000:50000 + 16000 * 16]
rawData = rawData[50000: 50000 + 200000]
print('rawData Length:', rawData.shape[0])
plt.plot(rawData)
plt.savefig(os.path.join('./', figDir, 'rawData.pdf'))
```

```
rawData Length: 200000
```

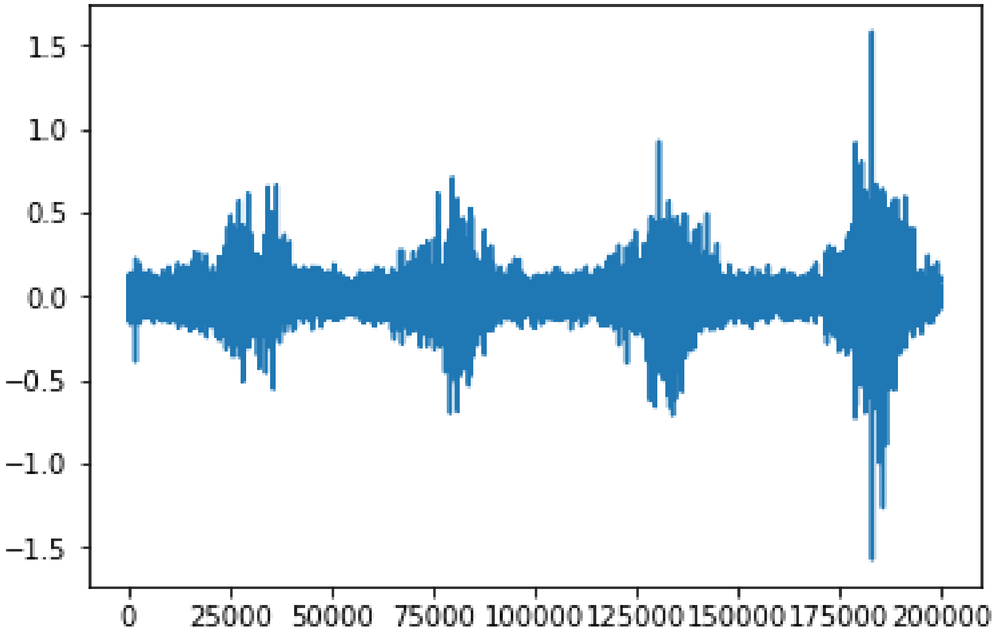


```
In [4]: #
        class config(object):
            ''''''
            fs = 16000

            ''' '''
            #
            order = 6
            #  cut-off
            high_cutoff = 100
            #  cut-off
            low_cutoff = 3000

            ''''''
            # 0.1s
            num_components = 100

            #
            winLen = int(rawData.shape[0] / num_components)

            #  num_components
            plot_rows = 10
            plot_cols = 10
            # plot_cols
            subplot_rows = 5
            subplot_cols = 2
```

```
    '''spike detection'''
    '''
        if fs == 16000:
            blank_time = 10
            spike_time = 80 // 75//
            total_time = 160//150//
        elif fs == 8000:
            blank_time = 5
            spike_time = 48
            total_time = 96
        elif fs == 4000:
            blank_time = 2
            spike_time = 32
            total_time = 64
    '''

    blank_time = 10
    spike_time = 80
    total_time = 160

    ''''''
    feature_select = 4 # 0:None 1:first-order deviation2:FFT, 3:wavelet, 4:auto-regressi
    # feature extraction

    # 4.auto-regression
    AR_order = 9
    # PCA
    pca_features = 0 # 0:pca pca

    '''num_clusters'''
    num_clusters_low = 6
    num_clusters_high = 8

config = config()
```

1 1.

In [8]:
```
#
#

from scipy.signal import butter, lfilter, freqz

def butter_highpass(cutoff, fs, order=5):
    nyq = 0.5 * fs
    normal_cutoff = cutoff / nyq
    b, a = butter(order, normal_cutoff, btype='high', analog=False)
```

```python
    return b, a

def butter_highpass_filter(data, cutoff, fs, order=5):
    b, a = butter_highpass(cutoff, fs, order=order)
    y = lfilter(b, a, data)
    return y

def butter_lowpass(cutoff, fs, order=5):
    nyq = 0.5 * fs
    normal_cutoff = cutoff / nyq
    b, a = butter(order, normal_cutoff, btype='low', analog=False)
    return b, a

def butter_lowpass_filter(data, cutoff, fs, order=5):
    b, a = butter_lowpass(cutoff, fs, order=order)
    y = lfilter(b, a, data)
    return y


# Filter requirements.
#

fs = config.fs       # sample rate, Hz
order = config.order
high_cutoff = config.high_cutoff
low_cutoff = config.low_cutoff  # desired low pass cutoff frequency of the filter, Hz 30

if low_cutoff > fs / 2:
    low_cutoff = 1

# 300 - 3000Hz

# Get the filter coefficients so we can check its frequency response.
b, a = butter_highpass(high_cutoff, fs, order)

print('b:', b, 'a:', a)

c, d = butter_lowpass(low_cutoff, fs, order)

# Plot the high pass frequency response.
plt.subplot(2,1,1)
w, h = freqz(b, a, worN=8000)
plt.plot(0.5*fs*w/np.pi, np.abs(h), 'b')
plt.plot(high_cutoff, 0.5*np.sqrt(2), 'ko')
plt.axvline(high_cutoff, color='k')
plt.xlim(0, 0.5*fs)
plt.title("Lowpass Filter Frequency Response")
plt.xlabel('Frequency [Hz]')
```

```
plt.grid()

# Plot the low pass frequency response.
plt.subplot(2,1,2)
w, h = freqz(c, d, worN=8000)
plt.plot(0.5*fs*w/np.pi, np.abs(h), 'b')
plt.plot(low_cutoff, 0.5*np.sqrt(2), 'ko')
plt.axvline(low_cutoff, color='k')
plt.xlim(0, 0.5*fs)
plt.title("Lowpass Filter Frequency Response")
plt.xlabel('Frequency [Hz]')
plt.grid()
```

```
b: [  0.92693687  -5.56162121  13.90405303 -18.53873737  13.90405303
  -5.56162121   0.92693687] a: [  1.          -5.84827464  14.25284067 -18.52806916  13.54992718
  -5.285636     0.85921196]
```

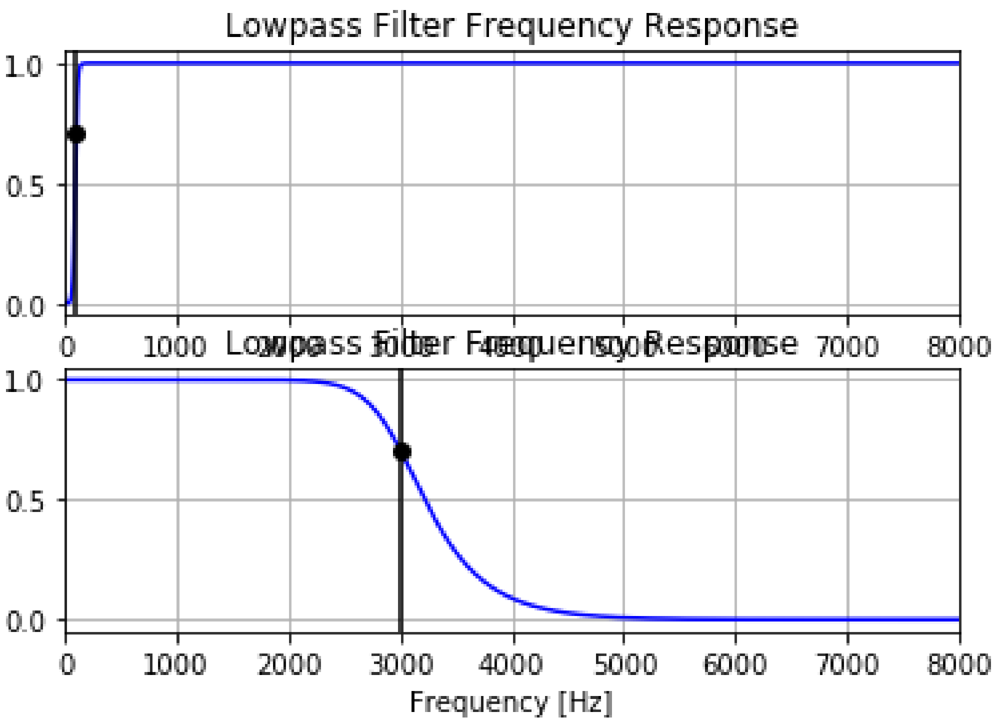


```
In [9]: #
        originalData = rawData

        #
        # high pass
```

```
filteredData = butter_highpass_filter(originalData, high_cutoff, fs, order)

if low_cutoff != 1:
    filteredData = butter_lowpass_filter(filteredData, low_cutoff, fs, order)

X_axis = np.arange(originalData.shape[0]) / config.fs

#plt.subplot(211)
plt.plot(X_axis, filteredData, color='r',label='Filtered', alpha=0.6)
plt.ylim((-2, 2))
#plt.subplot(212)
plt.plot(X_axis, originalData, color='b', linestyle='--', label='Original', alpha=0.5)
plt.ylim((-2, 2))
plt.xlim((0, X_axis[-1]))
plt.legend(loc=2, fontsize='17')
plt.xlabel('time(s)')
plt.ylabel('voltage(mv)')
plt.savefig(os.path.join('./', figDir, 'filtered.pdf'))

np.save('./filteredData.npy', filteredData)
```

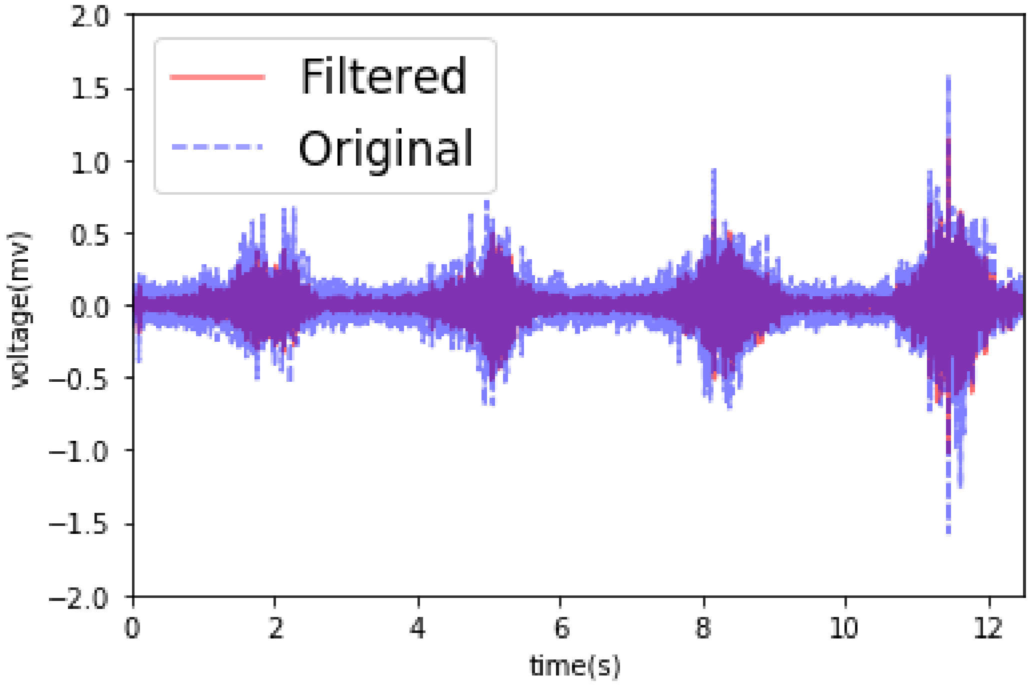

2 2. Independent component analysis

```
In [10]: # step 2 Independent Component Analysis
         from sklearn.decomposition import FastICA, PCA

         #
         num_components = config.num_components # 1600, 0.1s

         X = np.reshape(filteredData, (num_components, -1))

         X = np.transpose(X) # X

         ica = FastICA(n_components=num_components) # n_components X
         S_ = ica.fit_transform(X)  # Reconstruct signals
         A_ = ica.mixing_  # Get estimated mixing matrix

         X_ = np.dot(S_, A_.T)
```

3 3.spikes

S_ spike

```
In [12]: #  amplitude threshold

         # Unsupervised Spike Detection and Sorting with Wavelets and Superparamagnetic Clusteri
         deviation = np.median(np.abs(filteredData) / 0.6745)

         # obsolute_thresholdspike
         obsolute_threshold = 4.0 * deviation

         print('Amplitude Threshold:', obsolute_threshold)

         plt.plot(filteredData)
         plt.axhline(obsolute_threshold, color='r')
         plt.savefig(os.path.join('./', figDir, 'threshold.pdf'))
```

```
Amplitude Threshold: 0.123406113737
```

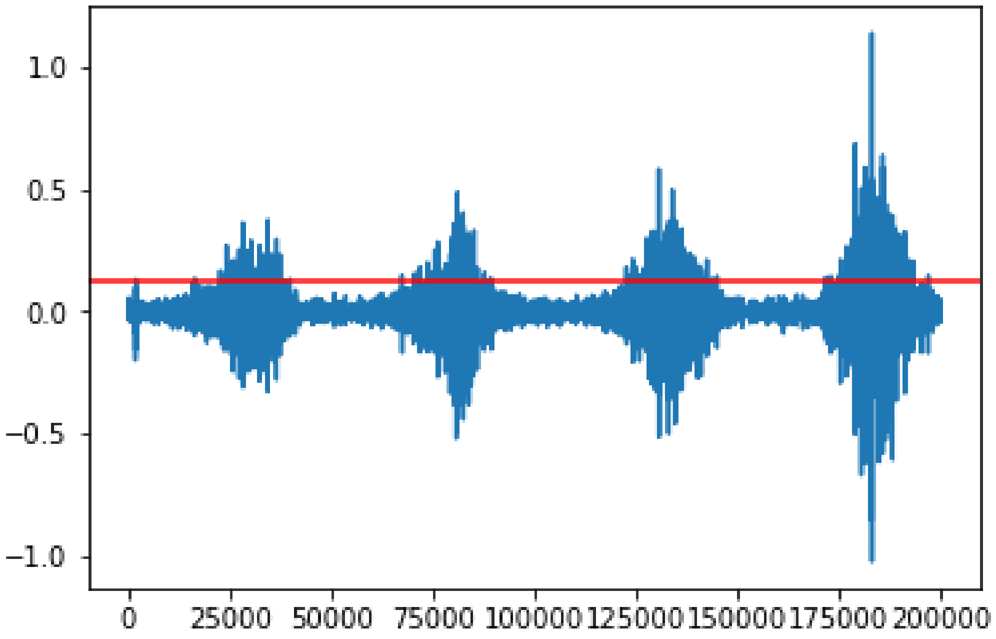


```
In [13]: # spike

         def spike_detect(x, free_threshold, amplitude_threshold, max_value_position, fixed_leng
             '''
             x  np.array
             free_threshold spike
             amplitude_threshold threhold
             fixed_length None
             ## x20
             '''
             max_value_index = max_value_position
             x_seq = x.flatten('C')
             times = x_seq.shape[0]
             spike = {}
             spike_index = 0

             i = 0
             while i < (times - free_threshold):
                 free_threshold_flag =[]

                 for j in range(free_threshold):
                     free_threshold_flag.append( np.abs(x_seq[i+j]) < amplitude_threshold )

                 free_threshold_flag.append(np.abs(x_seq[i+free_threshold]) > amplitude_threshol

                 if np.all(free_threshold_flag):
```

```
                    # free_threshold  amplitude_threshold,  free_threshold  amplitude_threshold

                    k = 1
                    while (i+free_threshold+k < times) and (x_seq[i+free_threshold+k] > amplitu
                        k = k + 1
                    # spike_max_id  aplitude_threshold
                    spike_max_id = i + free_threshold + np.argmax( np.abs(x_seq[i+free_threshol
                    # record the index of the spike maxima

                    # spike
                    if spike_max_id >=max_value_position:
                        spike[str(spike_index)] = [spike_max_id - max_value_index]

                        #
                        # spike
                        spike_end_id = spike_max_id + fixed_length - max_value_index
                        # spike
                        if spike_end_id < times:
                            spike[str(spike_index)].append( spike_end_id )

                        spike_index = spike_index + 1
                        i = spike_end_id


                i =  i + 1

            # dictcopy()
            # python 2.7
            for keys in spike.copy():
                if len(spike[keys]) != 2:
                    del spike[keys]

            return spike
```

In [14]:
```
spike_array = []

#
plot_rows = config.plot_rows
plot_cols = config.plot_cols

assert plot_rows * plot_cols == num_components

for i in range(plot_rows):
    plt.figure()
    for j in range(plot_cols):
        data = S_[:, i * plot_cols + j]

        spikes = spike_detect(data, config.blank_time, obsolute_threshold, config.spike
```

```python
    plt.subplot(config.subplot_rows, config.subplot_cols, j+1)
    plt.plot(data)
    plt.xticks([])
    plt.yticks([])
    plt.ylim([-0.2, 0.2])

    n = 0
    if spikes.has_key(str(n)):
        spike_start = spikes[str(n)][0]
        spike_end = spikes[str(n)][1]
        y = data[spike_start: spike_end]

        ## debug spike0
        maxIndex = np.argmax(np.abs(y))
        y = y * np.sign(y[maxIndex])


        spike_array.append(np.expand_dims(y, 0))
        x = np.array(list(range(spike_start, spike_start + y.shape[0], 1)))
        plt.plot(x, y, color='r')

    plt.savefig(os.path.join('./', figDir, 'ICA_spikes{}.pdf'.format(i)))
```

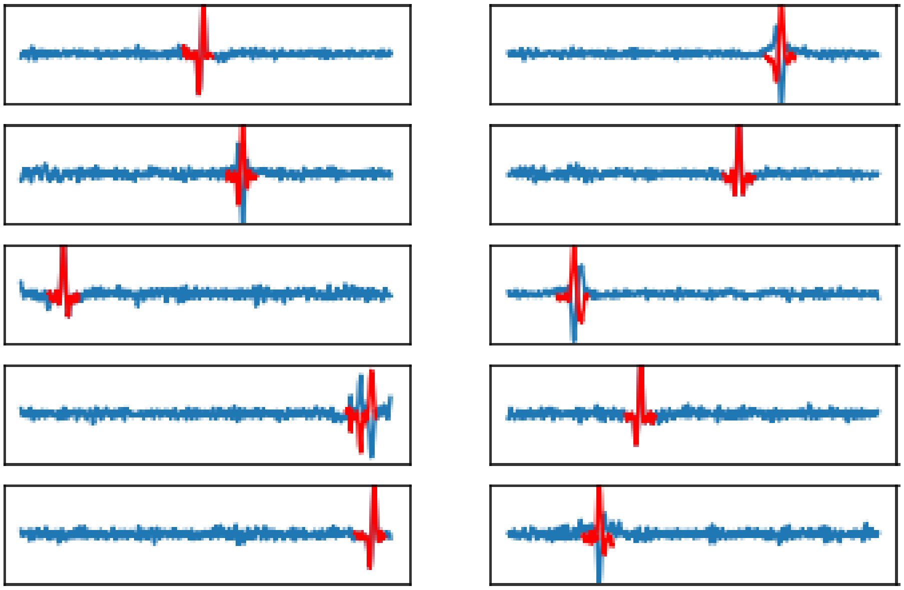

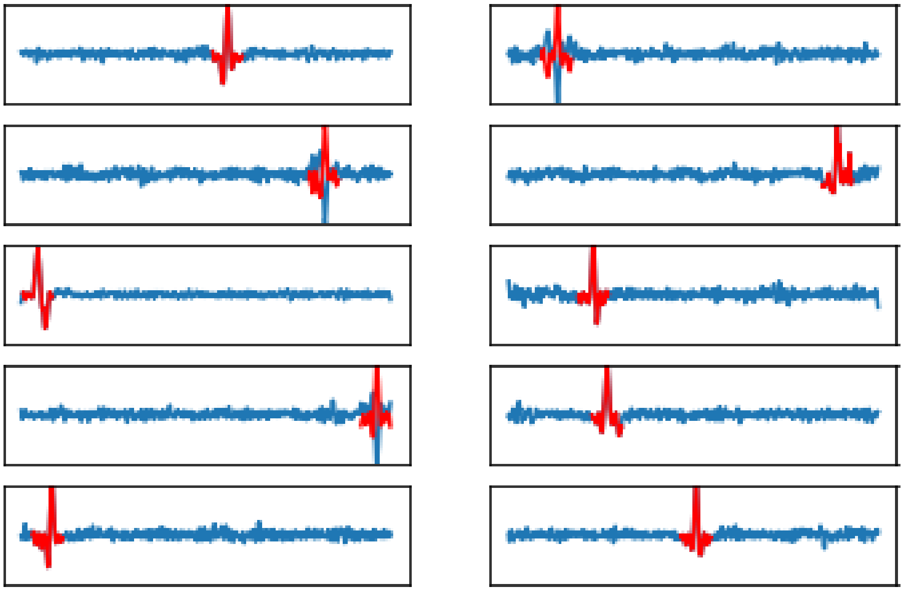

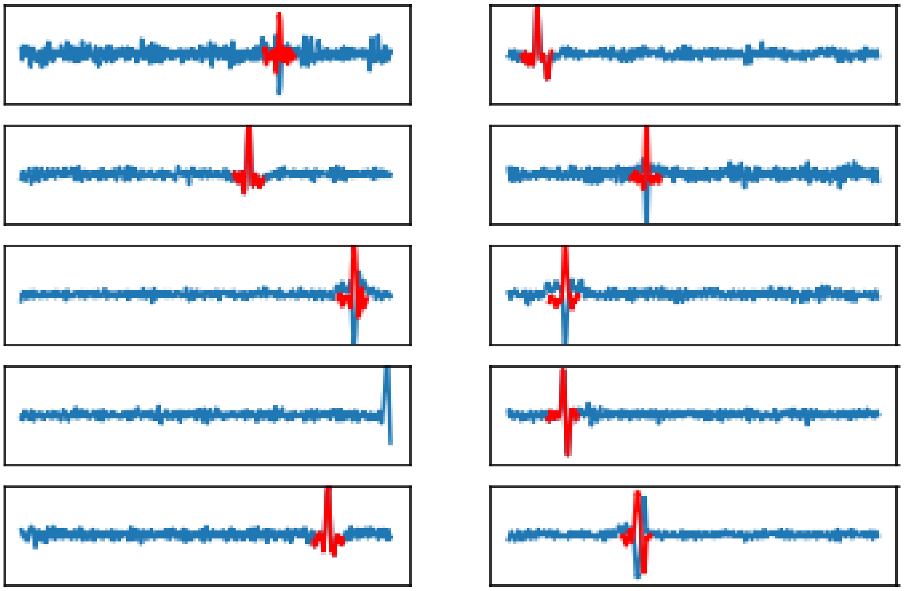

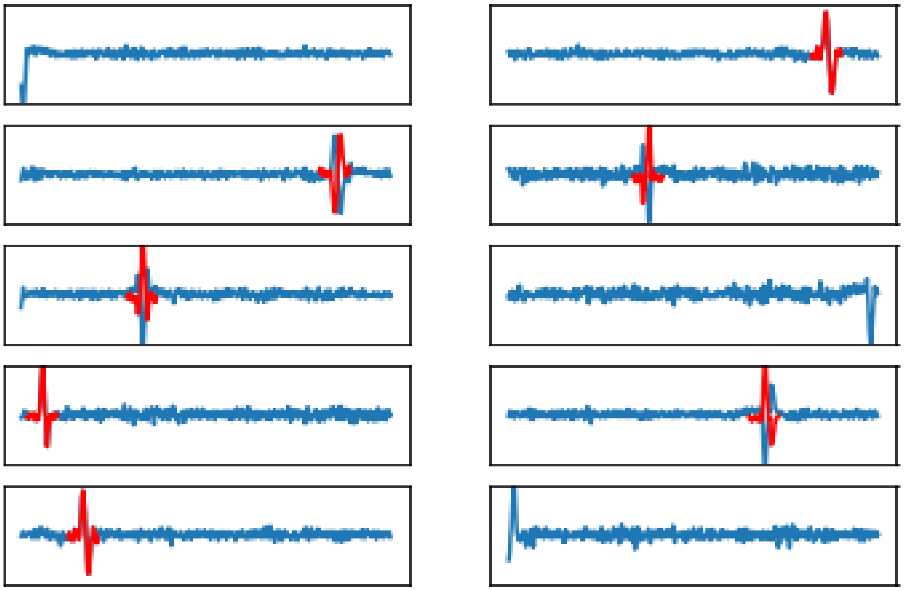

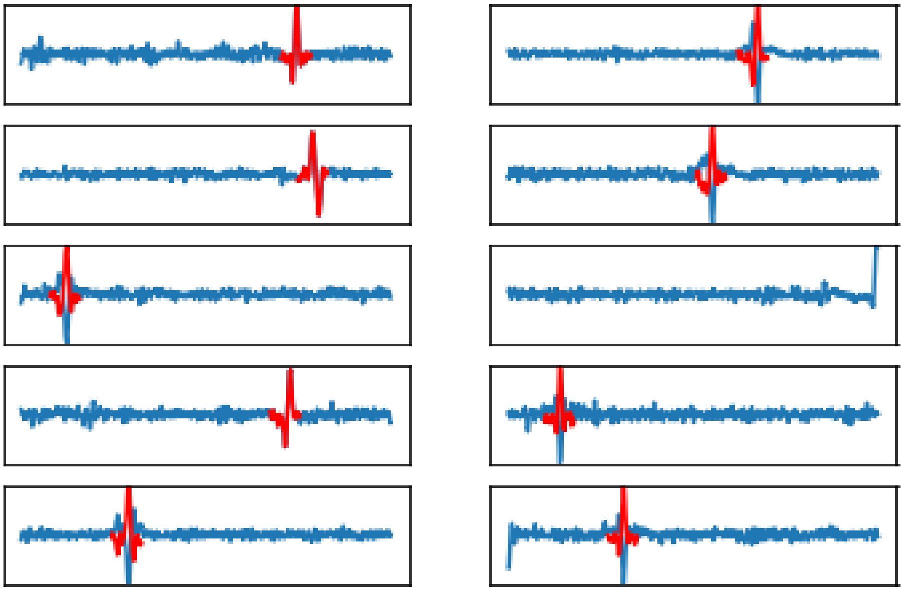

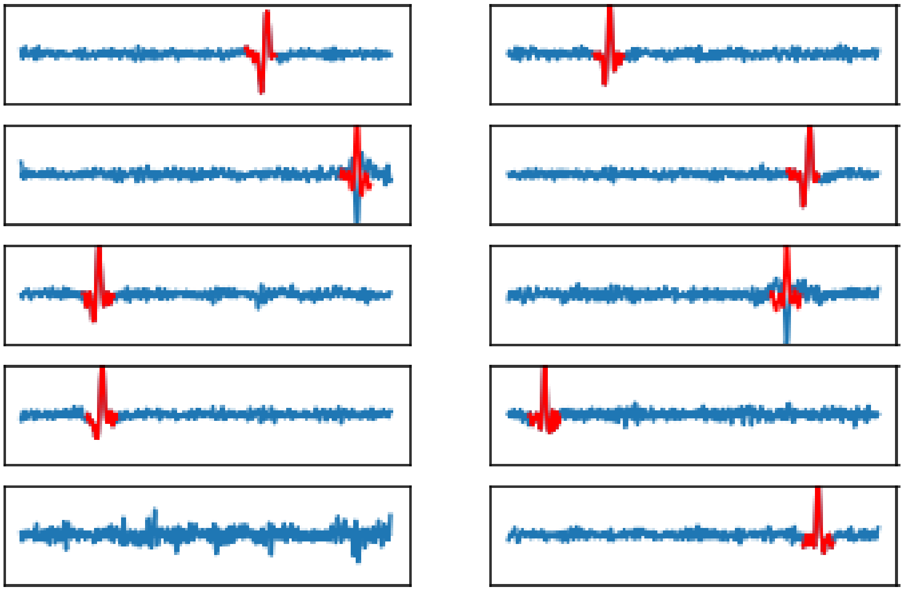

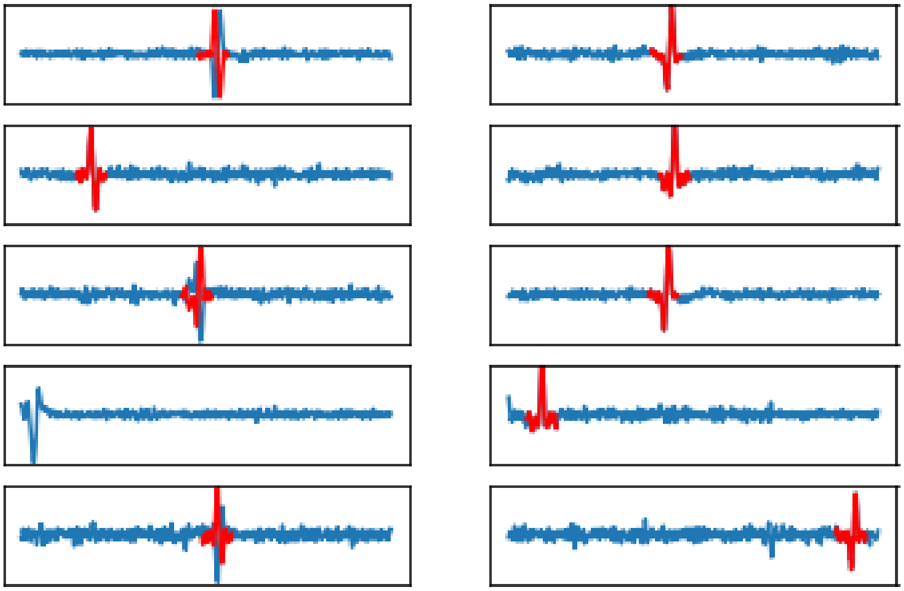

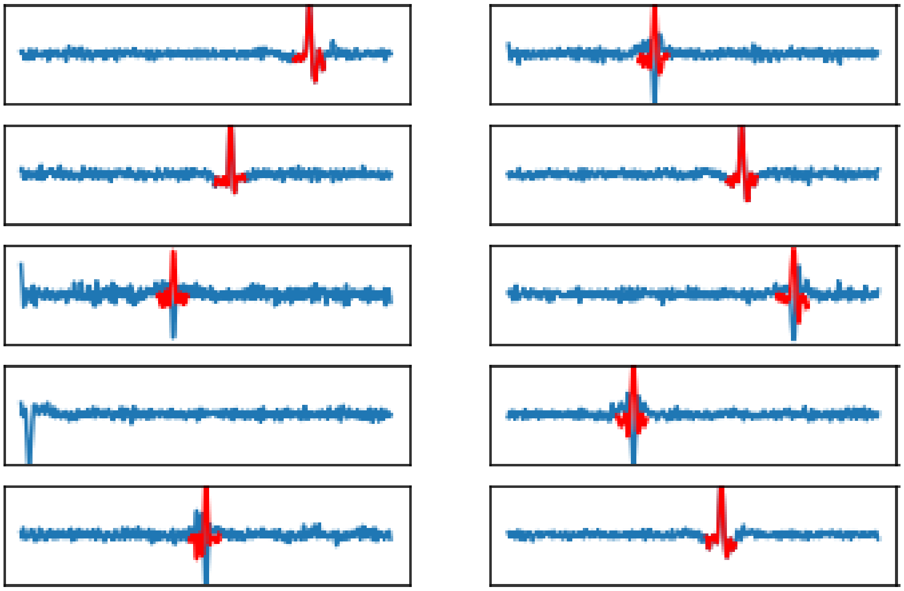

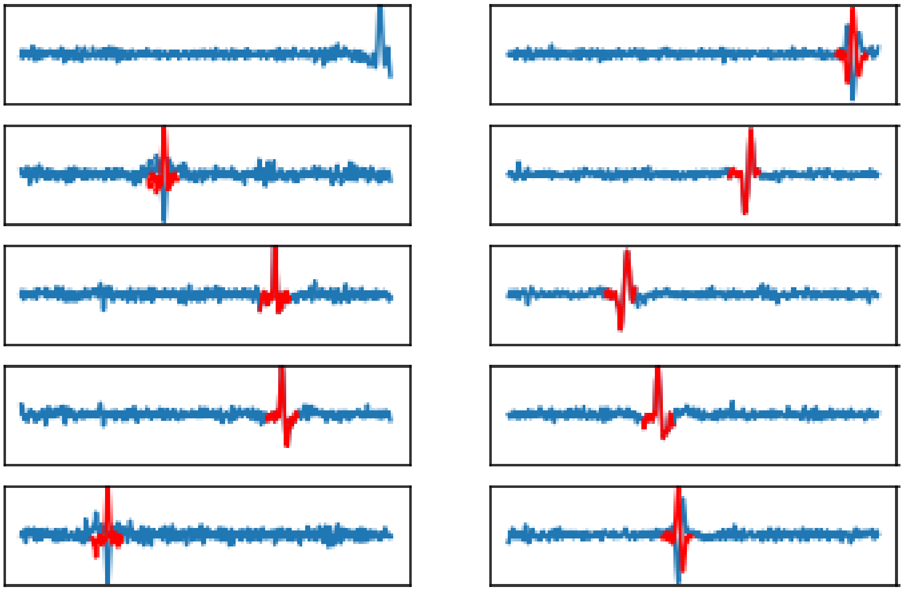

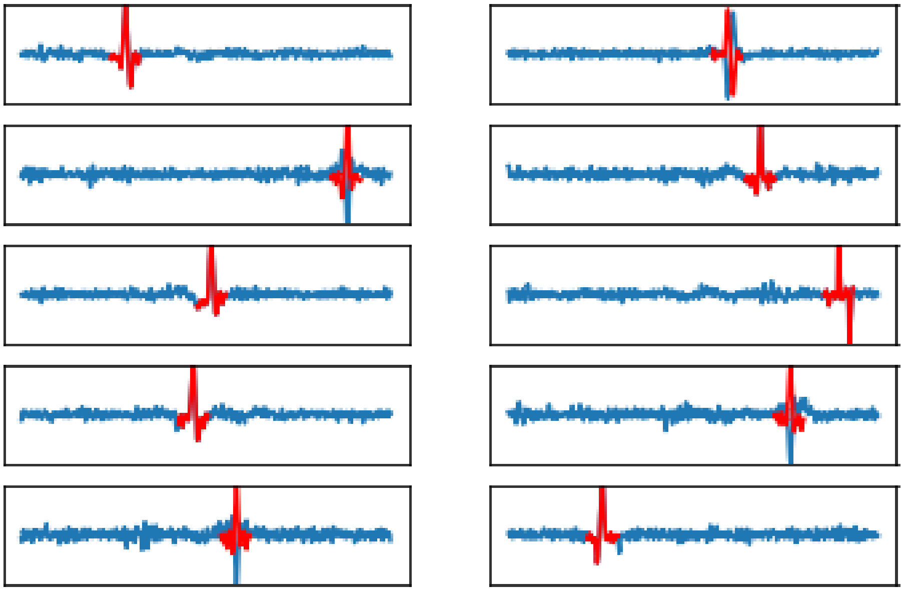

In [15]:
```
# auto-regression
def autoregression(timeSeq, M):
    N = timeSeq.shape[0]
    X = []
    y = []
    for i in range(N-M):
        rowVector = timeSeq[i:i+M]
        X.append(rowVector[::-1])
        y.append(timeSeq[i+M])
    X = np.array(X)
    y = np.array(y)
    # y
    y = np.expand_dims(y,axis=1)
    part1 = np.linalg.inv(np.matmul(np.transpose(X), X))
    part2 = np.matmul(np.transpose(X), y)
    a_opt = np.matmul(part1, part2)
    return a_opt

def generate_autoregressive_features(rawData, order):
    AR_features = []
    for i in rawData:
        AR_features.append(np.squeeze(autoregression(i, order)))
    return np.array(AR_features)
```

In [16]:
```
# sklearnspikes
# grid search
```

```python
from sklearn.cluster import KMeans
from sklearn import preprocessing

spike_array = np.vstack(spike_array)
X = spike_array
print('origin:', X.shape)

X_feature = None
## feature extraction
# 1.  unsupervised learning
if config.feature_select == 1:
    X_roll = np.roll(X, 1, axis=1)
    X_deriv = (X_roll - X)[:, 1:]
    X_feature = np.concatenate((X, X_deriv), axis=1)

#2. FFT
elif config.feature_select == 2:
    X_fft = np.fft.fft(X,n=None, axis=1)
    #X_fft = np.concatenate((X_fft.real, X_fft.imag), axis=1)
    X_fft = np.sqrt(np.square(X_fft.real) + np.square(X_fft.imag))
    X_feature = X_fft

#3. wavelet
elif config.feature_select == 3:
    X_wavelet = []
    widths = np.arange(1, 6)
    for i in range(X.shape[0]):
        cwtmatr = signal.cwt(X[i], signal.ricker, widths)
        cwtmatr = np.reshape(cwtmatr, (1,-1))
        X_wavelet.append(cwtmatr)
    X_wavelet = np.vstack(X_wavelet)
    X_feature = X_wavelet

#4. autoregressive
# ()   M
elif config.feature_select == 4:

    AR_order = config.AR_order
    X_ar = generate_autoregressive_features(X, AR_order)
    X_feature = X_ar

# features
else:
    X_feature = X

# PCA
from sklearn.decomposition import PCA
pca = None
```

```python
if config.pca_features != 0:
    pca = PCA(n_components=config.pca_features)
    X_feature = pca.fit_transform(X_feature)


scaler = preprocessing.StandardScaler().fit(X_feature)
X_scale = scaler.transform(X_feature)

#  grid search

## scoring
from sklearn.metrics import calinski_harabaz_score

def my_calinski_score(estimator, X, y=None):
    cluster_labels = estimator.predict(X)
    calinski_score = calinski_harabaz_score(X, cluster_labels)
    return calinski_score

from sklearn.metrics import silhouette_samples, silhouette_score
def my_silhouette_scoring(estimator, X, y=None):
    # cluster_labelscluster_labels
    #  n_clusters
    cluster_labels = estimator.predict(X)
    silhouette_score_avg = silhouette_score(X, cluster_labels)
    sample_silhouette_values = silhouette_samples(X, cluster_labels)
    num_clusters = estimator.get_params()['n_clusters']

    count_large_than_avg = 0
    for i in range(num_clusters):
        # np.array
        ith_cluster_silhouette_values = sample_silhouette_values[cluster_labels == i]
        mean_ith_cluster_silhouette_values = np.mean(ith_cluster_silhouette_values)
        if mean_ith_cluster_silhouette_values > silhouette_score_avg:
            count_large_than_avg = count_large_than_avg + 1

    return count_large_than_avg / num_clusters

from sklearn.model_selection import GridSearchCV

parameters = {'n_clusters':list(range(config.num_clusters_low,config.num_clusters_high)

# estimator
grid_search = GridSearchCV(estimator = KMeans(), param_grid = parameters, scoring = my_

grid_search = grid_search.fit(X_scale)

num_clusters = grid_search.best_params_['n_clusters']
kmeans = grid_search.best_estimator_
```

```
        clustering = kmeans

        print(num_clusters)
        #print(grid_search.cv_results_)
```

```
origin: (91, 160)
6
```

```
In [17]: '''
         color_maps = ['orange',
         'green',
         'red',
         'cyan',
         'black',
         'magenta',
         'yellow',
         'pink',
         'gray',
         'indigo',
         'chartreuse',
         'yellowgreen']
         '''


         color_maps = ['black',
         'gray',
         'silver',
         'snow',
         'rosybrown',
         'firebrick',
         'red',
         'darksalmon',
         'sienna',
         'sandybrown',
         'bisque',
         'tan',
         'moccasin',
         'gold',
         'darkkhaki',
         'lightgoldenrodyellow',
         'yellowgreen',
         'chartreuse',
         'darkgreen',
         'seagreen',
         'mediumspringgreen',
         'lightseagreen',
```

```
'paleturquoise',
'darkcyan'
]


label_all = np.zeros((num_components,1))
inverseFlag_all = np.zeros((num_components,1)) # spike A_
spike_dict = {} #sourcespike

for i in range(plot_rows):
    plt.figure()
    for j in range(plot_cols):
        data = S_[:, i * plot_cols + j]

        spikes = spike_detect(data, config.blank_time, obsolute_threshold, config.spike

        plt.subplot(config.subplot_rows, config.subplot_cols, j+1)

        n = 0
        if spikes.has_key(str(n)):
            spike_start = spikes[str(n)][0]
            spike_end = spikes[str(n)][1]
            y = data[spike_start: spike_end]

            ## debug spike0
            maxIndex = np.argmax(np.abs(y))
            inverseFlag = np.sign(y[maxIndex])
            y = y * inverseFlag
            inverseFlag_all[i * plot_cols + j] = inverseFlag

            # warning
            y_reshape = np.reshape(y, (1,-1))

            '''feature extraction'''
            #
            if config.feature_select == 1:
                y_reshape_roll = np.roll(y_reshape, 1, axis=1)
                y_reshape_deriv = (y_reshape_roll - y_reshape)[:, 1:]
                y_feature = np.concatenate((y_reshape, y_reshape_deriv), axis=1)

            # fft
            elif config.feature_select == 2:
                y_reshape_fft = np.fft.fft(y_reshape,n=None, axis=1)
                #y_reshape_fft = np.concatenate((y_reshape_fft.real, y_reshape_fft.imag
                y_reshape_fft = np.sqrt(np.square(y_reshape_fft.real) + np.square(y_res
                y_feature = y_reshape_fft

            # wavelet
```

```
        elif config.feature_select == 3:
            y_reshape_wavelet = signal.cwt(np.squeeze(y_reshape), signal.ricker, wi
            y_reshape_wavelet = np.reshape(y_reshape_wavelet, (1,-1))
            y_feature = y_reshape_wavelet

        # auto-regression
        elif config.feature_select == 4:
            y_reshape_ar = generate_autoregressive_features(y_reshape, config.AR_or
            y_feature = y_reshape_ar

        #
        else:
            y_feature = y_reshape

        # PCA
        if config.pca_features != 0:
            y_feature = pca.transform(y_feature)

        y_scale = scaler.transform(y_feature)
        y_scale = np.reshape(y_scale, (1,-1))
        label = int(clustering.predict(y_scale))
        score = clustering.score(y_scale)

        label_all[i * plot_cols + j] = label + 1
        # spike key=spike id, start,end,label
        spike_dict[str(i * plot_cols + j)] = [spike_start]
        spike_dict[str(i * plot_cols + j)].append(spike_end)
        spike_dict[str(i * plot_cols + j)].append(label)
        spike_dict[str(i * plot_cols + j)].append(score)

        x = np.array(list(range(spike_start, spike_start + y.shape[0], 1)))

        # debug0
        plt.plot(data * inverseFlag, color='blue')

        plt.plot(x, y, color=color_maps[label])
        plt.xticks([])
        plt.yticks([])
        plt.ylim([-0.2, 0.2])
    else:
        plt.plot(np.array(list(range(data.shape[0]))), data, color='blue')
        plt.xticks([])
        plt.yticks([])
        plt.ylim([-0.2, 0.2])

plt.savefig(os.path.join('./', figDir, 'ICA_spikes_cluster{}.pdf'.format(i)))
```

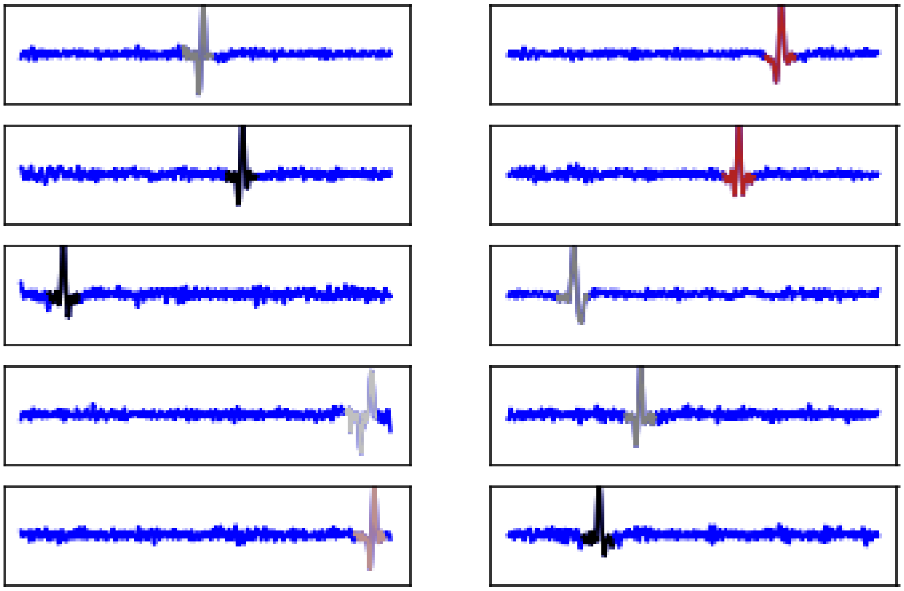

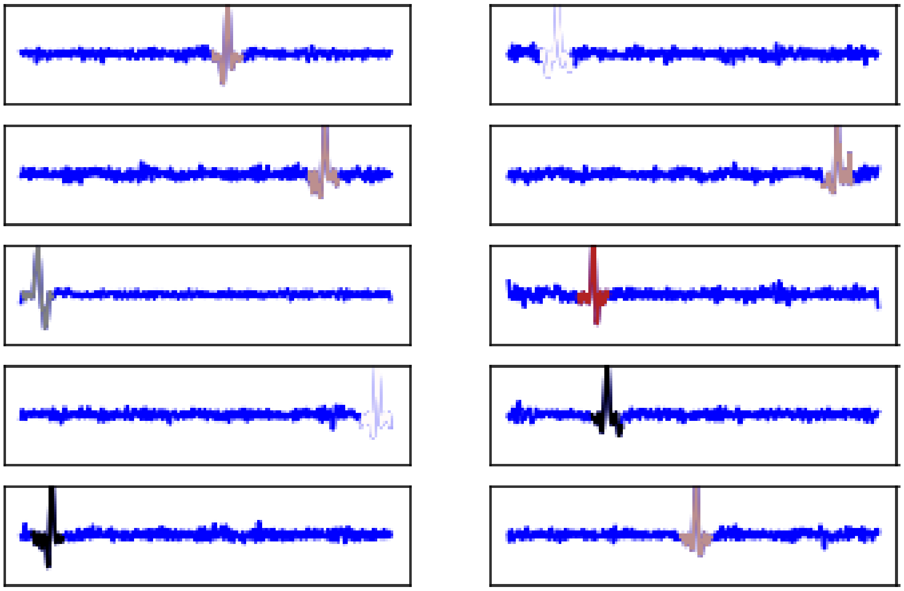

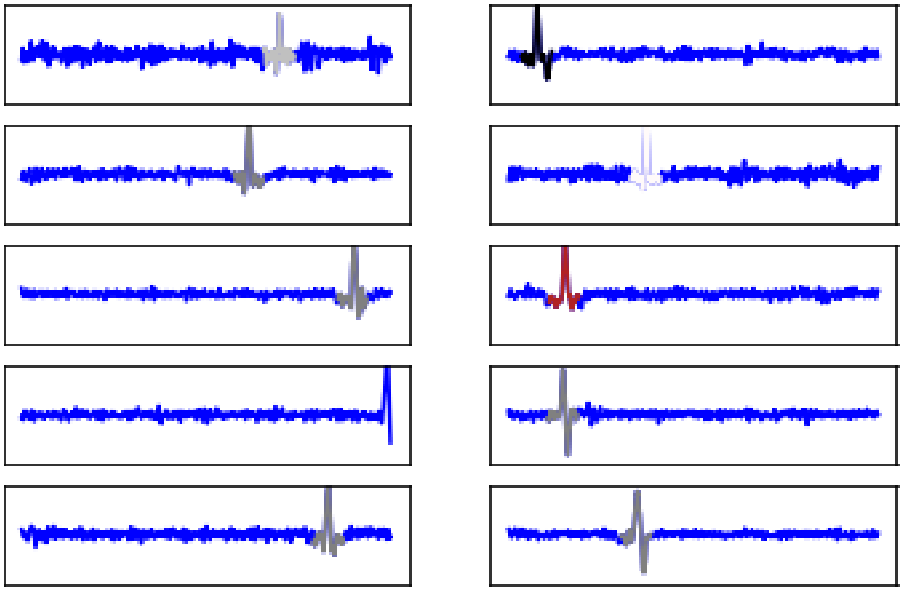

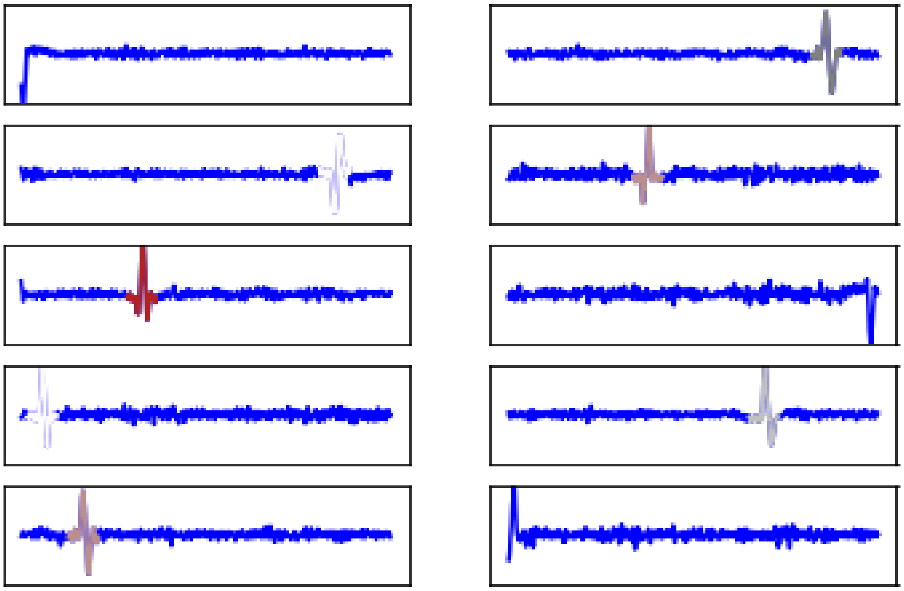

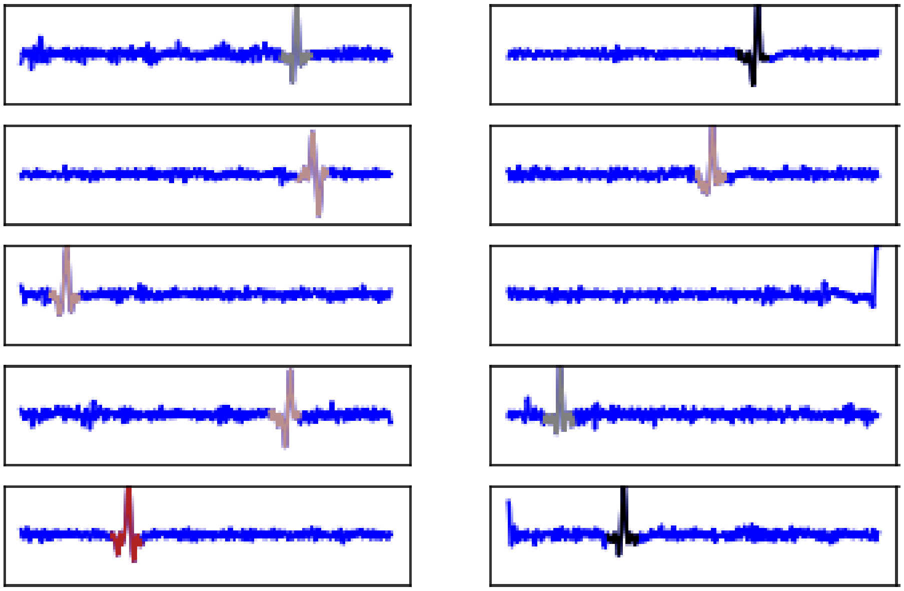

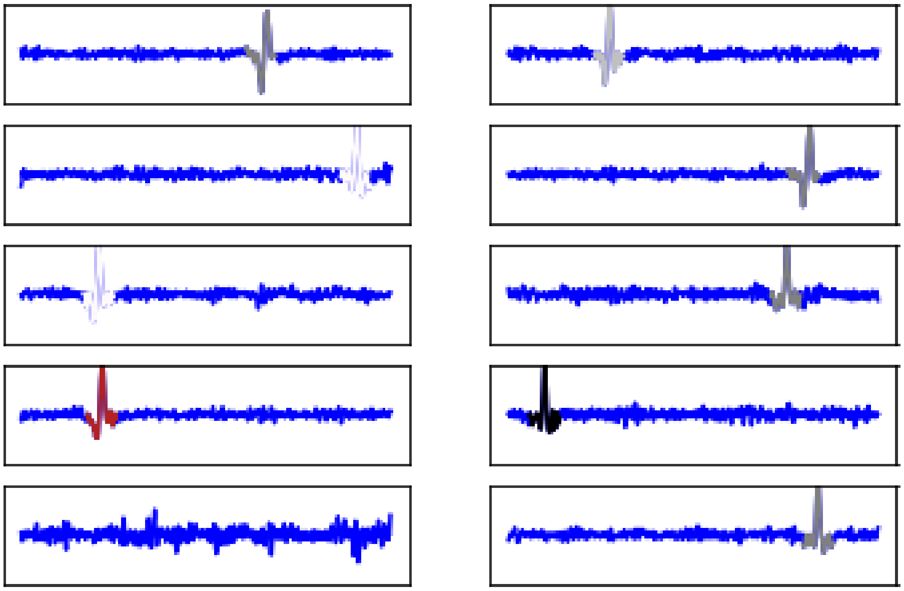

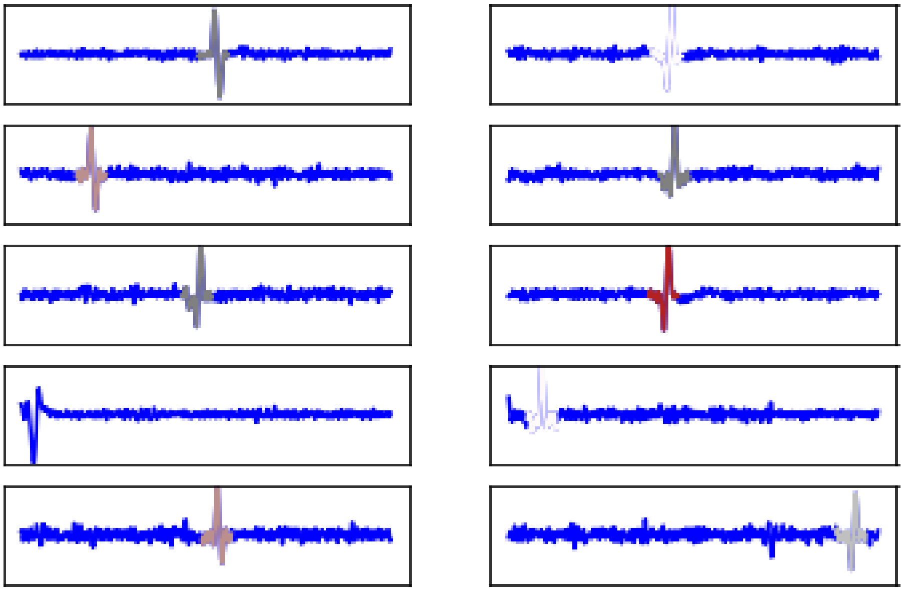

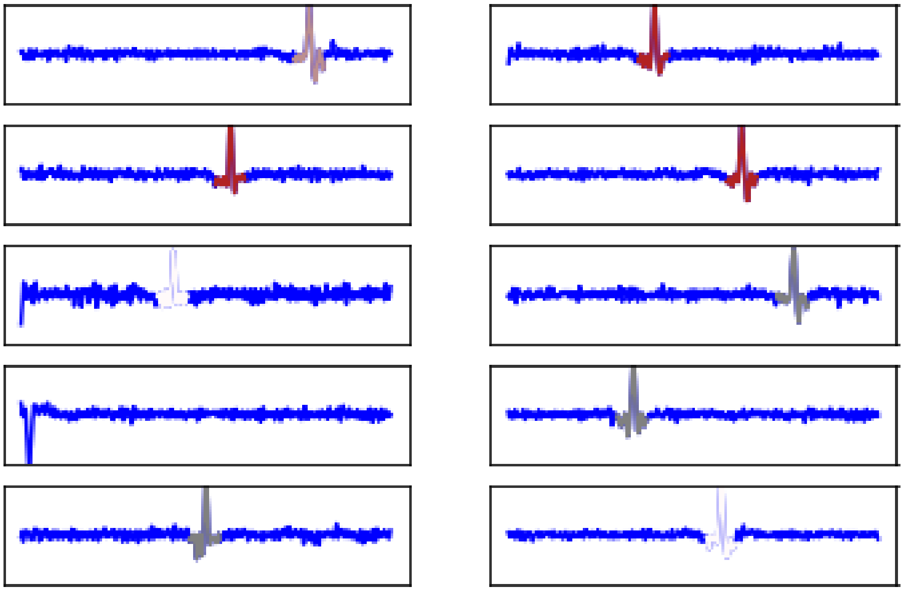

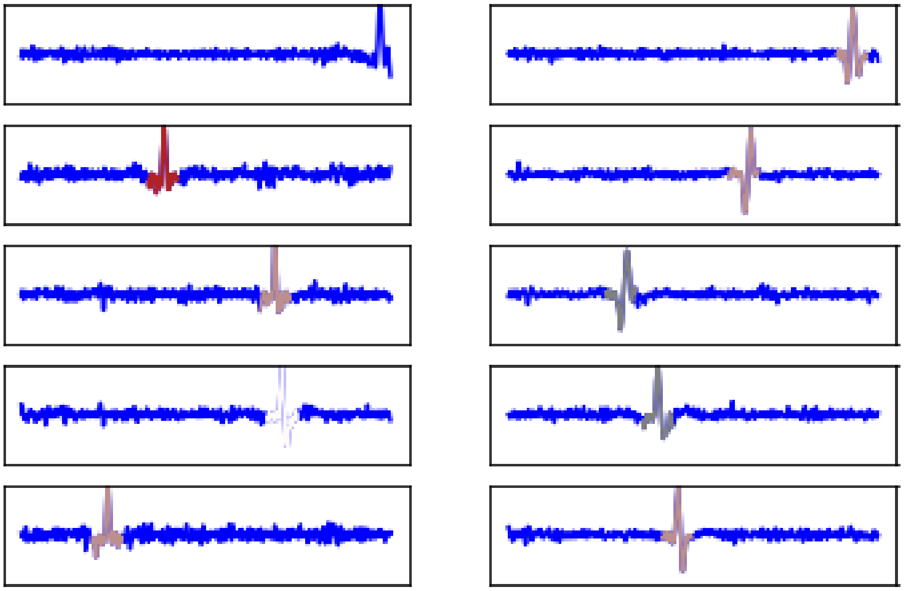

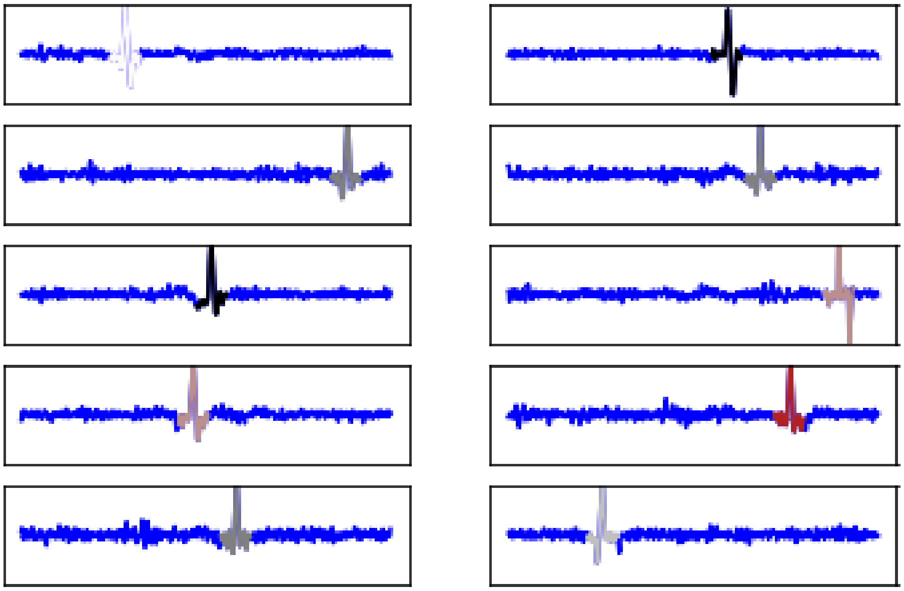

In [18]:
```
#
from mpl_toolkits.mplot3d import Axes3D
fig = plt.figure()
ax = Axes3D(fig, rect=[0, 0, .95, 1], elev=40, azim=6)
plt.cla() # clear the current axis
```

```
labels = clustering.predict(X_scale)

# PCA
pca3d = PCA(n_components=3)
X_3d = pca3d.fit_transform(X_scale)
#ax.scatter(X_3d[:, 2], X_3d[:, 0], X_3d[:, 1], c=labels.astype(np.float))
for i in range(np.shape(labels)[0]):
    ax.scatter(X_3d[i, 2], X_3d[i, 0], X_3d[i, 1], c=color_maps[labels[i] % len(color_m


ax.w_xaxis.set_ticklabels([])
ax.w_yaxis.set_ticklabels([])
ax.w_zaxis.set_ticklabels([])
plt.savefig(os.path.join('./', figDir, 'clusterings.pdf'))
```

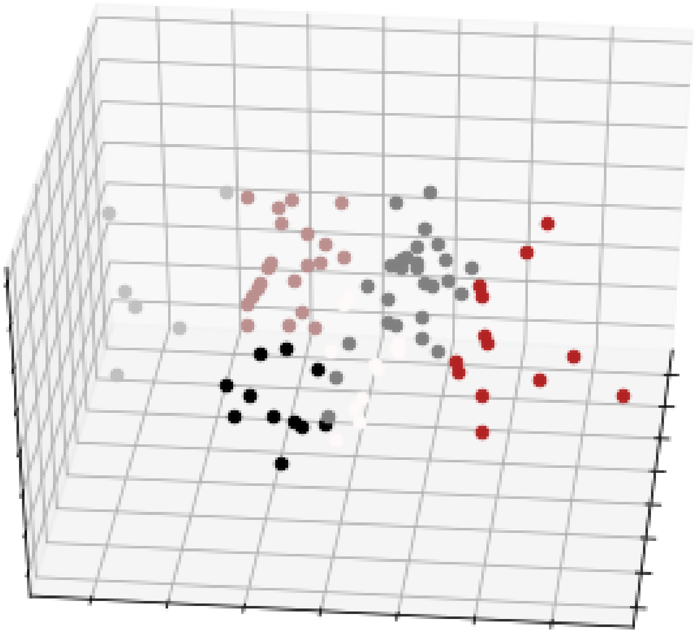

In [19]:
```
# label
count_clusters = np.zeros( num_clusters)
for i in range(0, num_components):
    if label_all[i] != 0:
        count_clusters[int(label_all[i] - 1)] = count_clusters[int(label_all[i] - 1)] +
```
